# Highly pathogenic PRRSV upregulates IL-13 production through nonstructural protein 9–mediated inhibition of N6-methyladenosine demethylase FTO

**DOI:** 10.1016/j.jbc.2024.107199

**Published:** 2024-03-18

**Authors:** Xingyu Gong, Yuan Liang, Jingjing Wang, Yipeng Pang, Fang Wang, Xiaohan Chen, Qiaoya Zhang, Chengchuang Song, Yanhong Wang, Chunlei Zhang, Xingtang Fang, Xi Chen

**Affiliations:** 1 Institute of Cellular and Molecular Biology, School of Life Science, Jiangsu Normal University, Xuzhou, China; 2 College of Veterinary Medicine, Qingdao Agricultural University, Qingdao, China; 3 Laboratory of Phylogenomics and Comparative Genomics, Jiangsu Normal University, Xuzhou, Jiangsu Province, China

**Keywords:** porcine reproductive and respiratory syndrome virus, interleukin-13, nsp9, fat mass and obesity associated (FTO) proteins, m^6^A

## Abstract

Porcine reproductive and respiratory syndrome virus (PRRSV), a highly infectious virus, causes severe losses in the swine industry by regulating the inflammatory response, inducing tissue damage, suppressing the innate immune response, and promoting persistent infection in hosts. Interleukin-13 (IL-13) is a cytokine that plays a critical role in regulating immune responses and inflammation, particularly in immune-related disorders, certain types of cancer, and numerous bacterial and viral infections; however, the underlying mechanisms of IL-13 regulation during PRRSV infection are not well understood. In this study, we demonstrated that PRRSV infection elevates IL-13 levels in porcine alveolar macrophages. PRRSV enhances m^6^A-methylated RNA levels while reducing the expression of fat mass and obesity associated protein (FTO, an m^6^A demethylase), thereby augmenting IL-13 production. PRRSV nonstructural protein 9 (nsp9) was a key factor for this modulation. Furthermore, we found that the residues Asp567, Tyr586, Leu593, and Asp595 were essential for nsp9 to induce IL-13 production *via* attenuation of FTO expression. These insights delineate PRRSV nsp9’s role in FTO-mediated IL-13 release, advancing our understanding of PRRSV's impact on host immune and inflammatory responses.

Porcine reproductive and respiratory syndrome virus (PRRSV), which belongs to the genus *Arterivirus* and family Arteriviridae, comprises a 15-kb–long single-stranded positive-sense RNA (1). It causes reproductive disorders and breathing difficulties in pigs, generating huge economic loss to the swine industry (2). The PRRSV genome contains 11 ORFs, encoding eight structural proteins and 16 nonstructural proteins (nsp), each possessing diverse functions in various steps of infection, replication, virulence, and virus–host interactions (3, 4, 5, 6, 7, 8). Immunosuppression after PRRSV infection may contribute to inefficient innate and acquired immune responses, modulating the expression and release of inflammatory and/or anti-inflammatory factors, upregulating immunosuppressive cytokines expression, and allowing the viruses to rapidly reproduce and cause disease (9, 10, 11, 12).

PRRSV infection has been reported to modulate various inflammatory factors and mediators, including cold-inducible RNA-binding protein, interleukin (IL)-17, IL-6, hypoxia-inducible factor 1a (HIF-1a), C1QBP, etc. (10, 11, 12, 13, 14, 15). The enzymatic domains of PRRSV nonstructural protein 1β (nsp1β) nuclease protease and papain-like cysteine protease have been noted to elevate HIF-1a expression, thus facilitating viral proliferation (11). Cold-inducible RNA-binding protein has been detected in stress granules as an RNA-binding protein, which positively regulates the NF-κB pathway to induce the expression of downstream proinflammatory cytokines, thus promoting inflammatory response during PRRSV infection (13). Furthermore, PRRSV-infected Marc-145 cells and porcine alveolar macrophages (PAMs) have been observed to exhibit increased C1QBP secretion and enhanced PRRSV-induced inflammatory cytokine production, including TNF-α, IL-1β, IL-6, IL-8, and MCP-1 production (10). PRRSV nsp2 can activate the NF-κB pathway, leading to the induction of NF-κB–dependent proinflammatory molecules, including IL-6, IL-8, COX-2, and RANTES (12, 13, 14, 15). Several studies have consistently reported that PRRSV infection causes an aberrant immune response called “cytokine storm,” in which the immune system of a healthy individual generates excessive amount of proinflammatory and immunosuppressive cytokines, severely overreacting to the viral infection (4, 12, 16). Accumulation of large amounts of proinflammatory cytokines can induce tissue injury and other serious inflammatory damage. The massive secretion of immunosuppressive cytokines negatively modulates innate immunity and can possibly lead to viral immune evasion. However, the mechanism underlying “cytokine storm” and immune suppression caused by PRRSV infection is unclear and requires further in-depth investigation.

IL-13, which belongs to the Th helper (Th) 2 family cytokines, is generally secreted by various cells, including alveolar macrophages, mast cells, Treg cells, activated T-cells, NK cells, innate lymphoid cells, and nonimmune cells (17, 18, 19, 20, 21, 22). It is involved in several physiological and pathological processes, particularly in inflammation, cancer, angiogenesis, immune suppression, and viral infection (17, 23). Increasing studies have revealed that IL-13 is an important mediator in multiple infectious and respiratory diseases, such as excessive inflammation, polarization of M2 macrophages, immune evasion of virus, pulmonary fibrosis, and viral pneumonia (24, 25, 26, 27). IL-13 has been noted to enhance Arg-1 expression, contributing to the increase in Th2 immune response, thus increasing the permissiveness of the host to *Trypanosoma cruzi* infection (28). During the development of liver cirrhosis and fibrosis in chronic hepatitis B virus infection, IL-13 has been reported to play a key role in linking metabolic with inflammatory components (29). Besides, activation of STAT6 expression has been observed to increase IL-13 production in Kaposi’s sarcoma herpesvirus–infected primary effusion lymphoma cell lines. This increase in IL-13 production represents a positive feedback loop potentiating proliferation of these cells through autocrine and/or paracrine effects (30). It has been reported that PRRSV infection causes statistically significant increase in anti-inflammatory and type I IFN-regulated gene expression, such as Mx1, IRF7, OAS1, IFNγ, IL-10, IL-13, IRF3, STING, OPN, IFNα, IFNβ, IL-2, and TNF-α (31, 32). In addition, infection of PRRSV alone in sows or in combination with *Mycoplasma hyopneumoniae* in piglets can induce alterations in immunoregulatory cytokines, such as IL-2, IL-10, IL-13, TGF-β1, TNF-α, CCL3L1, MIG, and PEPCAM-1 (33). IL-13 has been frequently observed to exhibit inflammatory roles in lung cancer and lung injury and can promote asthmatic airway inflammation, mucus overproduction, bronchial hyperresponsiveness, and immunoglobulin E synthesis (34). To date, although a few studies have shown that PRRSV could induce IL-13 release, the underlying reason and mechanism of this process are still unclear and need to be exhaustively investigated.

N6-Methyladenosine (m^6^A) is one of the most important posttranscriptional modifications of RNA and is highly conserved in size, structure, and function in various species, including mammals, plants, insects, and viruses (35, 36, 37). It is regulated by three groups of cellular proteins, namely, writers, including methyltransferase-like 3 (METTL3), methyltransferase-like 14 (METTL14), and Wilms’ tumor 1 associating protein; erasers, including α-ketoglutarate-dependent dioxygenase homolog 5 (ALKBH5) and fat mass and obesity associated protein (FTO), which eliminate m^6^A modification from RNAs; and readers, including YTH-domain containing family 1 (YTHDF1), YTHDF2, and YTHDF3, which regulate mRNA stability, translation, and cellular localization (38, 39). Recently, increasing studies have predicted that m^6^A methylation might be involved in controlling various cellular, pathological, and biological processes, such as stress response, innate and adaptive immune responses, immunosuppression, stem cell differentiation, cancer development, microRNA activities, and viral infection (38, 39, 40, 41, 42). Multiple viruses, such as HIV-1, hepatitis B and C virus (HBV), dengue virus, yellow fever virus, and severe acute respiratory syndrome coronavirus clade 2 (SARS-CoV-2), have been indicated to interact with m^6^A in the host genomic RNAs (35, 38, 39, 40, 42, 43). A previous study has reported that the host cell m^6^A methyltransferase, METTL3, decreased RIG-I binding and subsequently reduced the activation of inflammation pathways to regulate innate immune responses induced by SARS-CoV-2 virus infection (35). Furthermore, the 3ʹ-UTR of m^6^A sites of HIV-1 has been found to recruit cellular YTHDF m^6^A “reader” proteins to enhance both HIV-1 protein and RNA expression as well as viral replication in CD4+ T cells (43). Besides, methyltransferases (METTL3/14) of m^6^A have been noted to modify the 50 epsilon of HBV pgRNA to promote its interaction with core proteins to regulate its encapsulation (38). These findings indicate that the internal m^6^A modification and viral RNA interact during viral infections, thus suggesting that research on RNA modifications in PRRSV genome and their functional relevance to IL-13 expression during viral infection is crucial.

In this study, we defined the role of PRRSV-induced m^6^A level in regulating the host inflammatory factor and demonstrated that cellular m^6^A machinery could increase IL-13 expression induced by viral infection. Importantly, the viral proteins were examined individually for their ability to affect IL-13 production, and the results revealed that nsp9 enhanced IL-13 secretion by promoting FTO mRNA degradation. Furthermore, targeted mutations indicated that 551 to 600 amino acid residues (aa) as well as D567, Y586, L593, and D595 in nsp9 played important roles in inducing IL-13 secretion in PRRSV-infected PAMs through FTO-bound m^6^A modification. Taken together, our results revealed a novel mechanism of inflammatory reaction to viral infection *via* m^6^A modification, in which PRRSV nsp9 decreased FTO-bound increasing m^6^A-modified motifs of IL-13 secretion.

## Results

### PRRSV infection strongly induces IL-13 secretion with high lung inflammation

**Figure 1:**
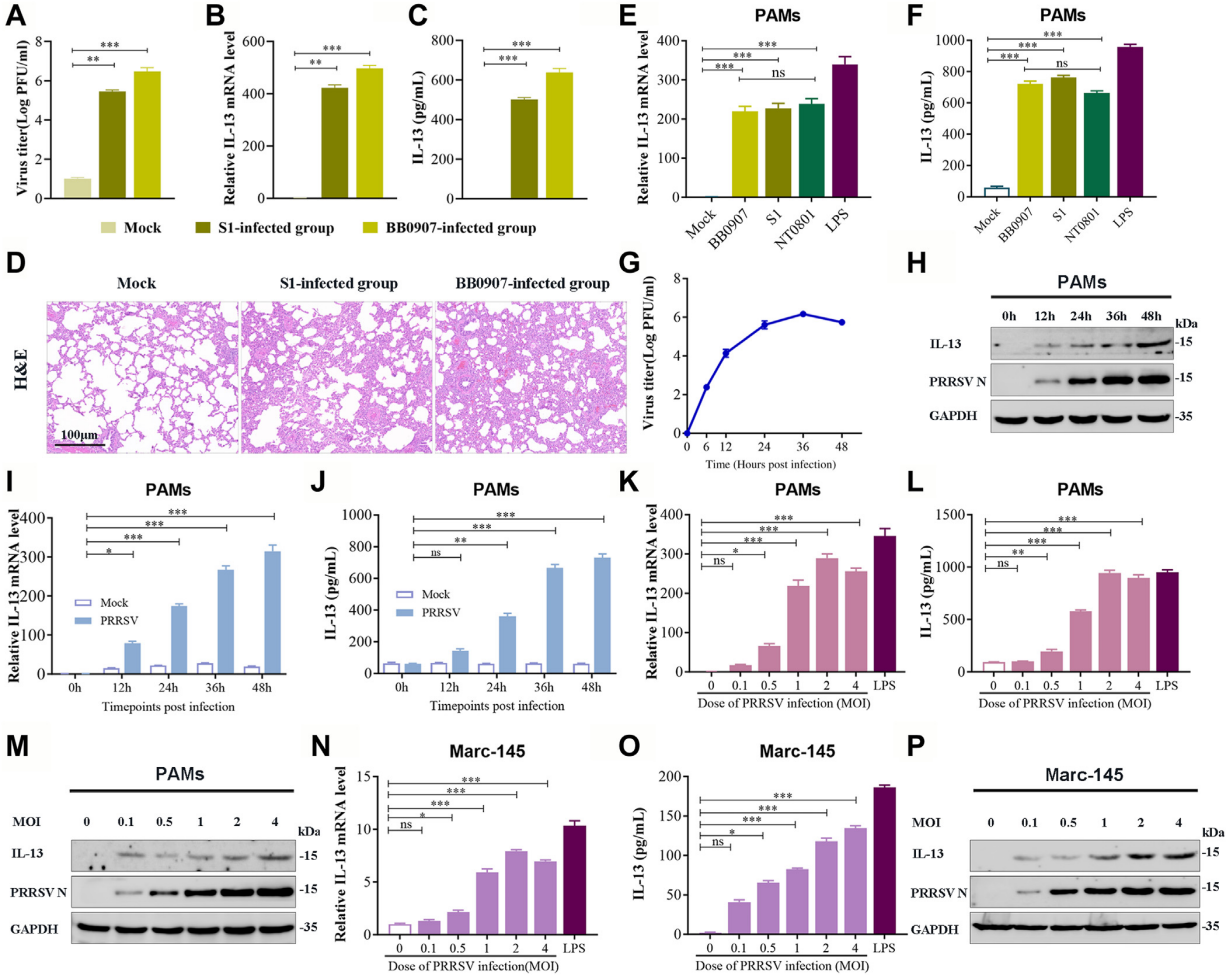
**PRRSV infection strongly induces IL-13 in PAMs.** The expression of IL-13 was detected in lung of pigs with PRSSV BB0907 or S1 strain infection. Virus titer (*A*) and IL-13 mRNA (*B*) in lungs were quantified by real-time PCR. *C*, the levels of IL-13 in lungs were quantified by ELISA. *D*, H&E staining of the lung samples. PAMs were cultured with or without inoculation of HP-PRRSV BB0907, C-PRRSV S1, and LP-PRRSV NT0801 at an MOI of 1. *E*, total RNA extracted from PAMs cell lysate at 36 h. Real-time PCR was used to analyze IL-13 expression (expressed as 2^−ΔΔCT^ values). GAPDH was used as reference gene. *F*, ELISA of IL-13 secretion in culture supernatants. *G*, TCID_50_ assay of the supernatants of infected PAMs to measure PRRSV infection kinetics. *H*–*J*, PAMs infected with PRRSV BB0907 (MOI of 1) for the indicated time. *H*, Western blot analysis of the protein levels of PRRSV N protein and IL-13. *I*, detection of IL-13 expression using RNA extracted from cell lysates by qRT-PCR. *J*, ELISA of IL-13 secretion in culture supernatant. *K*–*M*, PAMs infected with PRRSV at an MOI of 0.1, 0.5, 1, 2, and 4 for 36 h. *K*, analysis of IL-13 mRNA level by qRT-PCR. *L*, ELISA of IL-13 expression in culture supernatant. *M*, Western blot analysis of the levels of PRRSV N protein and IL-13. *N*–*P*, Marc-145 cells incubated with PRRSV BB0907 at an MOI of 1 for 36 h. *N*, IL-13 mRNA level, (*O*) IL-13 release, and (*P*) IL-13 protein level. All assays were repeated at least three times. Bars represent mean ± SD. ∗∗∗*p* < 0.001; ∗∗*p* < 0.01; ∗*p* < 0.05; ns, not significant. IL-13, Interleukin-13; MOI, multiplicity of infection; PAM, porcine alveolar macrophage; PRRSV, porcine reproductive and respiratory syndrome virus; TCID_50_, 50% tissue culture infective dose.

In our study, lung tissue samples from pigs infected with PRRSV S1 or BB0907 strain were analyzed. Quantitative PCR was utilized to determine PRRSV viral titers and IL-13 levels in the collected lung tissues. Our findings revealed an elevation in IL-13 levels in both S1 and BB0907 strain-infected tissues, with a correlation observed between higher IL-13 levels and increased viral loads (Fig. 1, *A*–*C*). Additionally, H&E staining of lung tissue sections revealed more pronounced pulmonary inflammation in tissues with higher IL-13 levels (Fig. 1*D*). To examine the effect of PRRSV infection on host cells’ production of IL-13, PAMs were inoculated with various PRRSV strains of differing virulence, including HP-PRRSV BB0907, C-PRRSV S1, and LP-PRRSV NT0801. Lipopolysaccharide-treated cells served as a positive control. All the three PRRSV strains significantly increased IL-13 mRNA expression (Fig. 1*E*) and IL-13 release level (Fig. 1*F*). Figure 1, *H*–*J* shows the obvious increase in IL-13 production at different time points following PRRSV infection; the IL-13 production rapidly increased from 12 to 36 hpi but moderately increased from 36 to 48 hpi, with PRRSV titers in infected PAMs reaching a peak at 36 hpi (Fig. 1*G*), suggesting that IL-13 induction was dependent on PRRSV replication.

To optimize viral infection, PAMs were infected with PRRSV at varying multiplicity of infection (MOI) of 0.1, 0.5, 1, 2, or 4. As shown in Figure 1, *K*–*M*, the protein level and expression of IL-13 mRNA were significantly higher in cells infected with PRRSV at an MOI of 1, when compared with those in cells infected at an MOI of 0.5. These results indicated that IL-13 production increased in PRRSV-infected cells in a dose-dependent manner. As PRRSV can grow in cultured Marc-145 cells (derived from embryonic African green monkey kidney tissue), Marc-145 cells were infected with PRRSV at a range of MOIs. The results of Western blot analysis, qRT-PCR, and ELISA revealed that PRRSV infection dramatically increased IL-13 mRNA expression (Fig. 1*N*) and release (Fig. 1*P*), as well as IL-13 protein (Fig. 1*O*), irrespective of the cell type.

### PRRSV infection upregulates cellular RNA m^6^A levels

**Figure 2:**
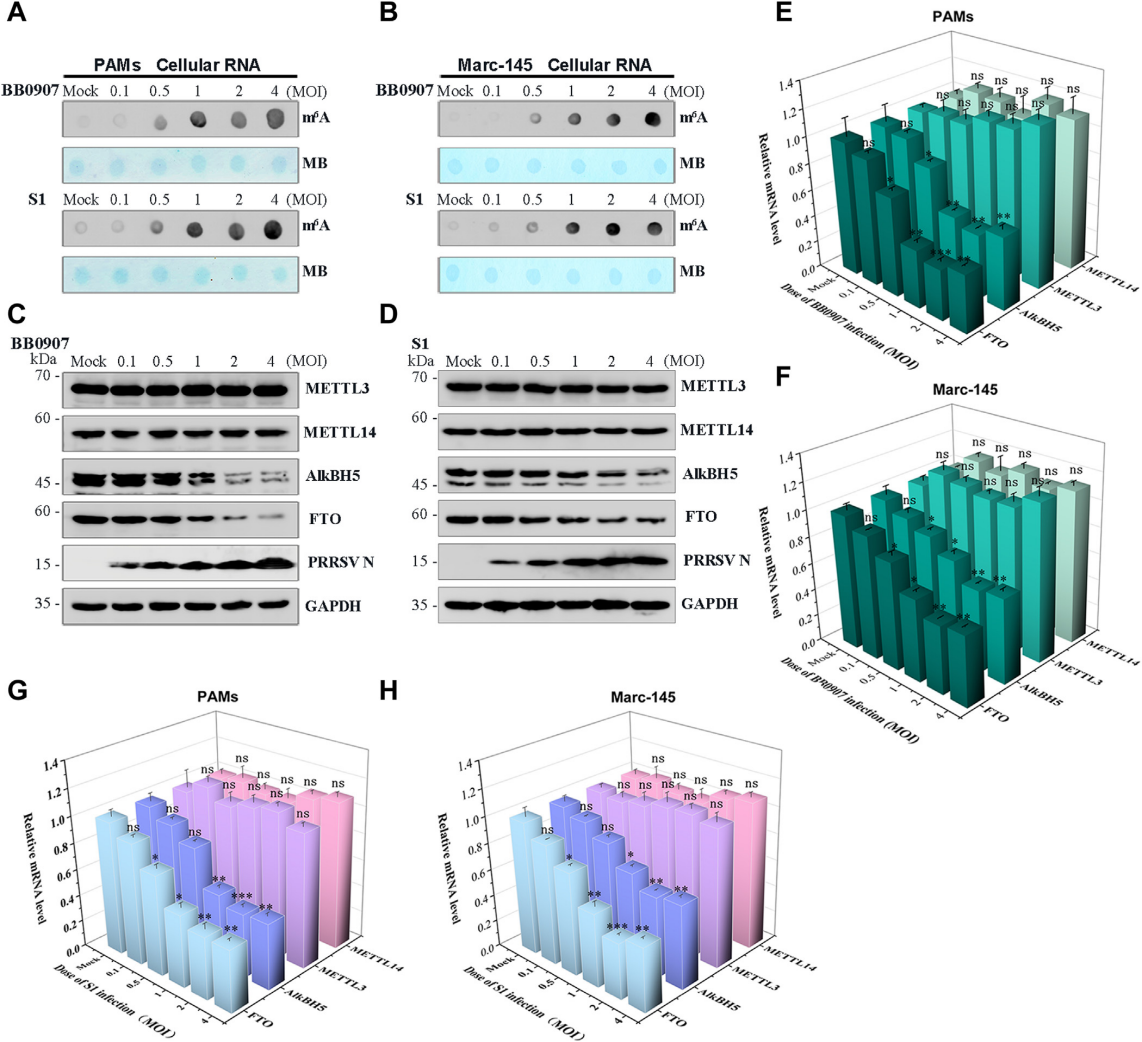
**PRRSV infection upregulates cellular RNA m**^**6**^**A levels in PAMs.***A*, PAMs or (*B*) Marc-145 cells infected with PRRSV BB0907 or S1 strain at the indicated MOI for 36 h. Relative m^6^A levels in total RNA (500 ng) collected from infected or control cells were measured by m^6^A dot blot analysis. MB staining was used as an RNA loading control. *C* and *D*, Western blot analysis of cell lysates from (*C*) BB0907-infected or (*D*) S1-infected samples to detect m^6^A writers (METTL3 and METTL14) and erasers (AlkBH5 and FTO). GAPDH was used as a loading control. *E* and *F*, qRT-PCR detection of the mRNA levels of METTL3, METTL14, AlkBH5, and FTO in BB0907-infected PAMs (*E*) and Marc-145 cells (*F*). *G* and *H*, qRT-PCR detection of the mRNA levels of METTL3, METTL14, AlkBH5, and FTO in S1-infected PAMs (*G*) and Marc-145 cells (*H*). Data represent mean ± SD of three independent experiments. ∗∗∗*p* < 0.001; ∗∗*p* < 0.01; ∗*p* < 0.05; ns, not significant, when compared with control samples as indicated. m6A, N6-Methyladenosine; MB, methylene blue; METTL, methyltransferase-like; MOI, multiplicity of infection; PAM, porcine alveolar macrophage; PRRSV, porcine reproductive and respiratory syndrome virus.

To systematically analyze the effects of PRRSV infection on cellular RNA m^6^A levels, PAMs and Marc-145 cells were infected with PRRSV BB0907 or S1 strain at a range of MOIs (0.1, 0.5, 1, 2, and 4, respectively). Dot blot assay was employed to measure the m^6^A levels in the total RNA from the control or PRRSV-infected cells at 36 hpi. The total RNA was stained with methylene blue (MB) as a loading control to normalize the relative m^6^A levels. When compared with the control cells, the cellular RNA m^6^A levels increased in both BB0907- and S1-infected PAMs (Fig. 2*A*) or Marc-145 cells (Fig. 2*B*) with increasing MOIs. To investigate the underlying mechanism of PRRSV-induced increase in cellular RNA m^6^A levels, we examined the expression of m^6^A writers (METTL3 and METTL14) and erasers (AlkBH5 and FTO) in BB0907- or S1-infected PAMs or Marc-145 cells (Fig. 2, *C*–*H*). In both BB0907- and S1-infected cells, no significant change in protein and mRNA levels of writers (METTL3 and METTL14) was observed after PRRSV infection (Fig. 2, *C*–*H*); however, the expressions of AlkBH5 and FTO were decreased in the PRRSV-infected group, with increasing m^6^A accumulation (Fig. 2, *C*–*H*). Accordingly, we selected the highly virulent HP-PRRSV BB0907 strain for subsequent experiments. Furthermore, as the increased cellular RNA m^6^A level reached a possible threshold at MOI of 1, MOI of 1 was used in the subsequent experiments. These results indicated that AlkBH5- and FTO-mediated enhancement of RNA m^6^A level during PRRSV infection occurred in a dose-dependent manner.

### AlkBH5 does not influence the expression level of IL-13

**Figure 3:**
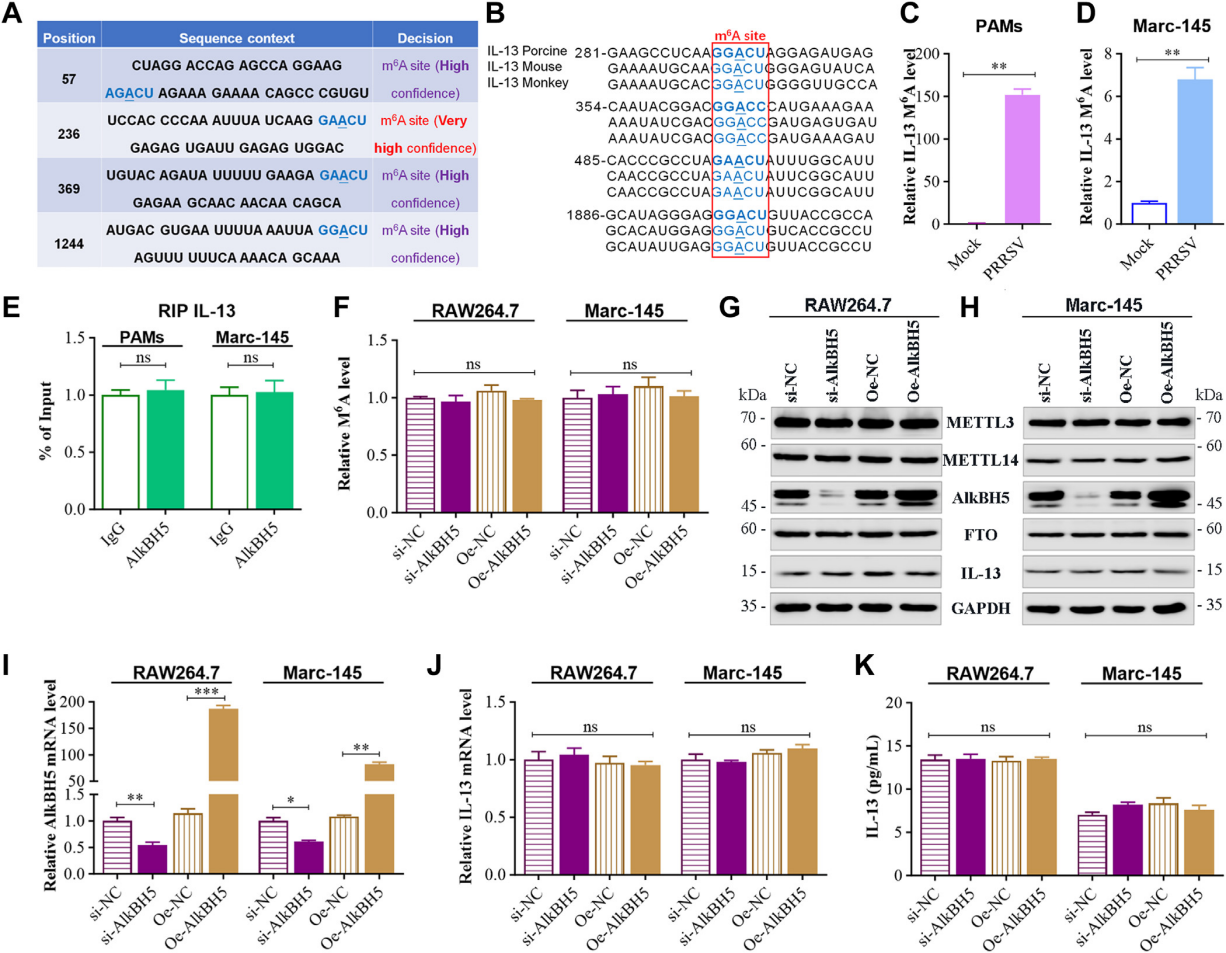
**AlkBH5 does not influence IL-13 production.***A*, IL-13 mRNA m^6^A modification sites predicted by SRAMP (http://www.cuilab.cn/sramp/). *B*, sequence comparison of IL-13 mRNA m^6^A modification sites in porcine, monkey, and human, corresponding to 281 nt, 354 nt, 485 nt, and 1886 nt of porcine. *C* and *D*, m^6^A-RIP-qPCR analysis of m^6^A enrichment on IL-13 mRNA in PRRSV-infected (*C*) PAMs and (*D*) Marc-145 cells. *E*, RIP-qPCR assay of the enrichment of AlkBH5 on the IL-13 mRNA m^6^A region. Marc-145 and RAW264.7 cells transfected with si-NC, si- AlkBH5, Oe-NC, and Oe- AlkBH5. *F*, IL-13 mRNA m^6^A level in Marc-145 and RAW264.7 cells determined by MeRIP-qPCR using anti-m^6^A antibody. *G* and *H*, protein level of m^6^A writers (METTL3 and METTL14), m^6^A erasers (AlkBH5 and FTO), and IL-13 in (*G*) RAW264.7 cells and (*H*) Marc-145 cells determined by Western blot analysis. *I* and *J*, the mRNA level of (*I*) AlkBH5 and (*J*) IL-13 in cells detected by qRT-PCR. *K*, IL-13 secretion ascertained by ELISA of cell supernatant. Data are expressed as mean ± SD, representative of three independent experiments. ∗∗∗*p* < 0.001; ∗∗*p* < 0.01; ∗*p* < 0.05; ns, not significant. IL-13, Interleukin-13; m6A, N6-Methyladenosine; METTL, methyltransferase-like; PAM, porcine alveolar macrophage; PRRSV, porcine reproductive and respiratory syndrome virus; RIP, RNA immunoprecipitation.

In our investigation into whether cellular RNA m^6^A upregulation impacts IL-13 expression, we initially used a sequence-based m^6^A modification site predictor, SRAMPA (http://www.cuilab.cn/sramp/), to identify potential methylation sites (Fig. 3*A*). This study involved Marc-145 cells, RAW264.7 cells, and PAMs, leading us to compare the sequences of IL-13 mRNA m^6^A modification sites across pigs, mice, and monkeys. The analysis showed that these sites are conserved in IL-13 mRNA at nucleotide positions 281, 354, 485, and 1886 in pigs (Fig. 3*B*). Following this, PAMs and Marc-145 cells were infected with PRRSV at an MOI of 1 for 36 h, and the IL-13 m^6^A levels were quantified using methylated RNA immunoprecipitation (MeRIP)-qPCR. Compared to the control, the PRRSV-infected samples exhibited a significant increase in IL-13 m^6^A levels (*p* < 0.01) (Fig. 3, *C* and *D*). Given the observed decrease in AlkBH5 and FTO expression due to PRRSV infection, we first probed whether AlkBH5 directly binds to IL-13 transcripts using RNA immunoprecipitation (RIP)-qPCR, which no alteration in IL-13 transcripts within the m^6^A region (Fig. 3*E*). We then assessed AlkBH5's effect on IL-13 expression by transfecting Marc-145 and RAW264.7 cells with either AlkBH5 knockdown or overexpression plasmids. Subsequent analysis through MeRIP-qPCR, qRT-PCR, ELISA, and Western blot showed no significant changes in IL-13 m^6^A levels (Fig. 3*F*), IL-13 protein levels (Fig. 3, *G* and *H*), mRNA expression (Fig. 3*J*), or secretion (Fig. 3*K*) in the siRNA-targeted AlkBH5 (si-AlkBH5) group, despite a reduction in AlkBH5 expression (Fig. 3*I*). Additionally, IL-13 production levels in the AlkBH5 overexpression (Oe-AlkBH5) group were unaffected by increased AlkBH5 expression, compared to the negative control overexpression (Oe-NC) group (Fig. 3, *G*–*K*), and the protein expression of METTL3, METTL14, and FTO remained largely unchanged. This evidence suggests that AlkBH5 does not influence IL-13 expression levels.

### FTO directly affects IL-13 production during the progression of PRRSV infection

**Figure 4:**
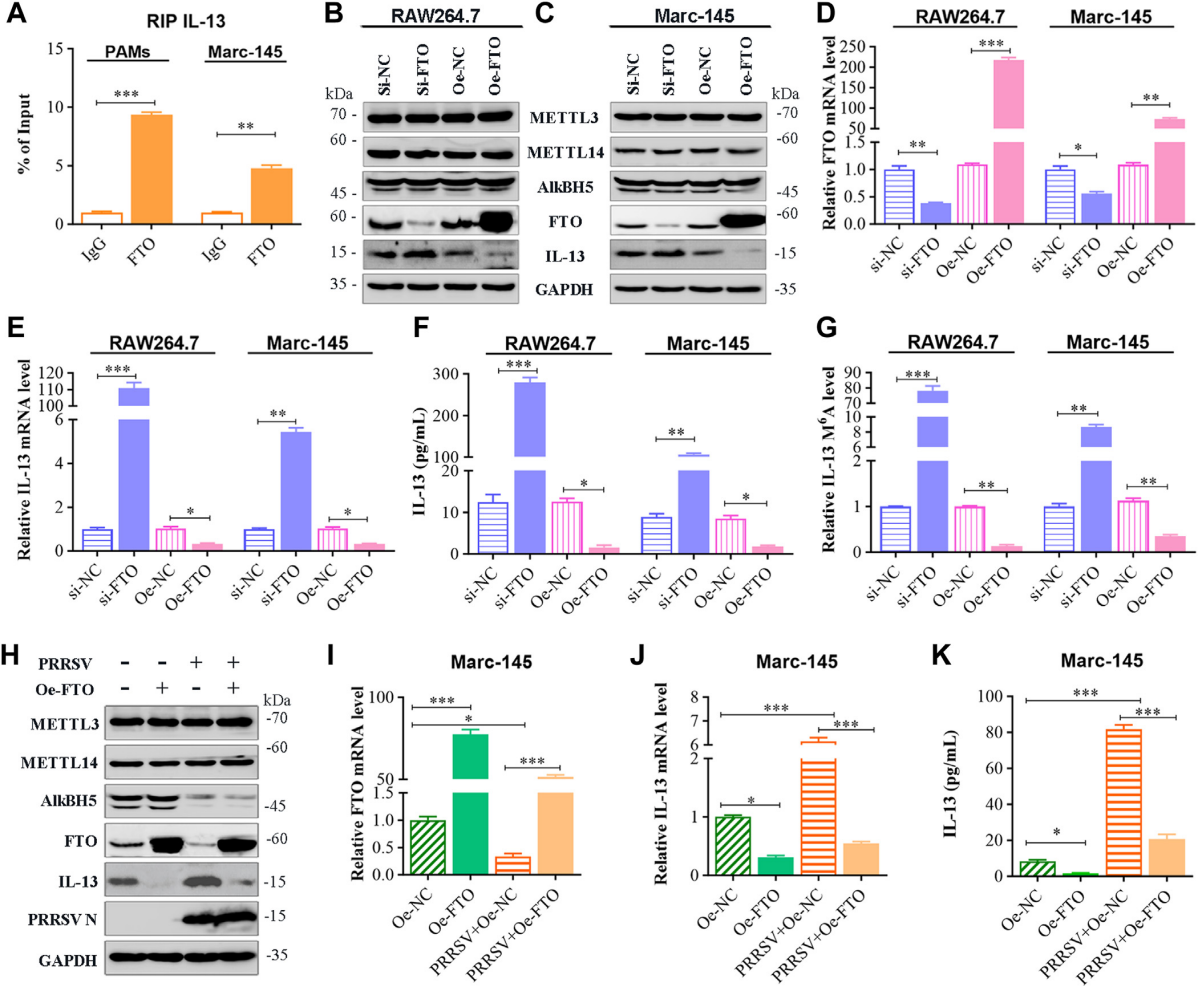
**FTO directly affects IL-13 production during the progression of PRRSV infection.***A*, RIP-qPCR assay of the enrichment of FTO on the IL-13 mRNA m^6^A region. Marc-145 and RAW264.7 cells transfected with si-NC, si-FTO, Oe-NC, and Oe-FTO, respectively. *B* and *C*, Western blot analysis for protein level of METTL3, METTL14, AlkBH5, FTO, and IL-13 in (*B*) RAW264.7 cells and (*C*) Marc-145 cells. *D* and *E*, the mRNA level of (*D*) FTO and (*E*) IL-13 in cells detected by qRT-PCR. *F*, IL-13 secretion ascertained by ELISA of cell supernatant. *G*, IL-13 mRNA m^6^A level in RAW264.7 and Marc-145 cells determined by MeRIP-qPCR using anti-m^6^A antibody. *H*–*K*, Marc-145 cells uninfected or infected with PRRSV at an MOI of 1 and co-transfected with or without Oe-FTO. *H*, Western blot for METTL3, METTL14, AlkBH5, FTO, IL-13, PRRSV N. *I* and *J*, the mRNA expression of (*I*) FTO and (*J*) IL-13 evaluated by qRT-PCR. *K*, IL-13 release detected by ELISA. Data are expressed as mean ± SD, three experiments. Significance. ∗∗∗*p* < 0.001; ∗∗*p* < 0.01; ∗*p* < 0.05; ns, not significant. IL-13, Interleukin-13; m6A, N6-Methyladenosine; METTL, methyltransferase-like; MOI, multiplicity of infection; PRRSV, porcine reproductive and respiratory syndrome virus; RIP, RNA immunoprecipitation; si-FTO, FTO-targeted siRNA.

To ascertain whether FTO directly binds to IL-13 transcripts, we employed RIP-qPCR. The results from FTO RIP-qPCR demonstrated a significant enrichment of IL-13 transcripts in the m^6^A region (Fig. 4*A*). To explore FTO's impact on IL-13 expression, we transfected FTO knockdown or overexpression plasmids into Marc-145 and RAW264.7 cells. Subsequent qRT-PCR, ELISA, and Western blot analysis revealed that, compared to the negative control siRNA (si-NC) group, IL-13 protein levels (Fig. 4, *B* and *C*), mRNA expression (Fig. 4*E*), and secretion (Fig. 4*F*) were significantly increased in the FTO-targeted siRNA (si-FTO) group, despite a decrease in FTO expression (Fig. 4, *B*–*D*). Conversely, in the FTO overexpression (Oe-FTO) group, compared to the Oe-NC group, IL-13 production was significantly reduced as FTO expression increased (Fig. 4, *B*–*F*), while the protein expression of METTL3, METTL14, and AlkBH5 remained largely unchanged.

Further analysis revealed alterations in IL-13 m^6^A levels in RAW264.7 and Marc-145 cells post-FTO knockdown or overexpression. The IL-13 m^6^A level rose in the si-FTO group compared to the si-NC group, while it significantly decreased in the Oe-FTO group compared to the Oe-NC group (Fig. 4*G*). These results suggest that FTO binds directly to IL-13 transcripts in an m^6^A-dependent manner. To further verify if FTO mediated IL-13 production during PRRSV infection, Marc-145 cells were inoculated with or without PRRSV (MOI of 1) and with or without Oe-FTO. The PRRSV+Oe-NC group showed significantly higher IL-13 mRNA expression and secretion (Fig. 4, *J* and *K*) but lower production of m^6^A erasers protein FTO (Fig. 4, *H* and *I*), compared to the Oe-NC group. In contrast, IL-13 production in the PRRSV+Oe-FTO group was inhibited due to the restored expression of FTO (Fig. 4, *H*–*K*), compared to the PRRSV+Oe-NC group. These findings underscore the role of FTO in regulating IL-13 expression during PRRSV infection.

### PRRSV viral proteins affect m^6^A levels

**Figure 5:**
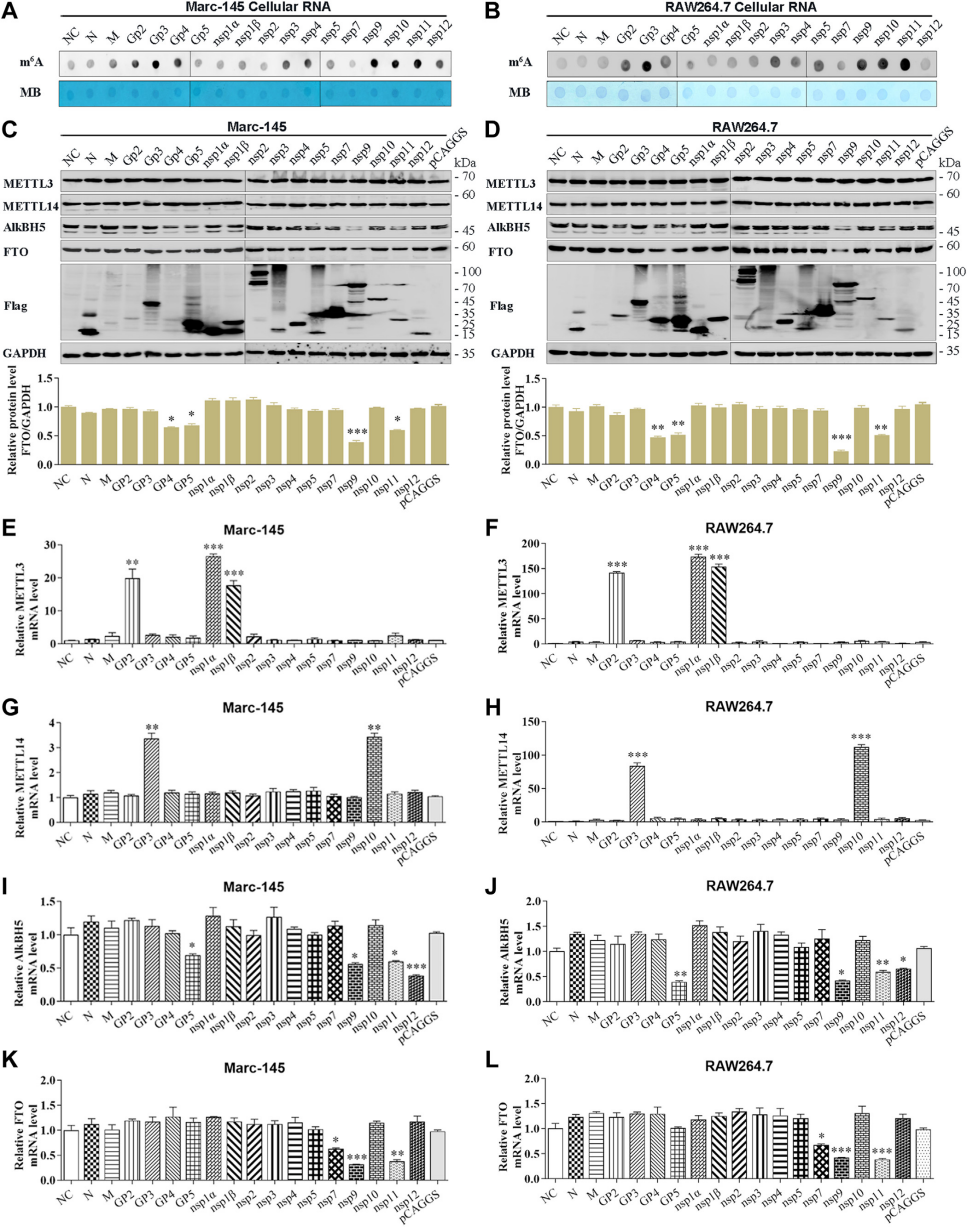
**PRRSV viral proteins affect m**^**6**^**A levels.***A* and *B*, dot blot assay of the m^6^A levels in (*A*) Marc-145 cells and (*B*) RAW264.7 cells. *C* and *D*, Western blot analysis of the protein levels of METTL3, METTL14, AlkBH5, FTO, and PRRSV N protein in (*C*) Marc-145 cells and (*D*) RAW264.7 cells. *E*–*L*, qRT-PCR detection of the mRNA expression of (*E* and *F*) METTL3, METTL14, (*I* and *J*) AlkBH5, and (*K* and *L*) FTO in Marc-145 cells and RAW264.7 cells. All data were compared with the NC group for at least three times, with each experiment performed in triplicate. Bars represent mean ± SD. ∗∗∗*p* < 0.001; ∗∗*p* < 0.01; ∗*p* < 0.05; ns, not significant. m6A, N6-Methyladenosine; METTL, methyltransferase-like; PRRSV, porcine reproductive and respiratory syndrome virus.

To identify viral proteins with the ability to increase m^6^A level, we constructed a series of recombinant plasmids by using the pCAGGS vector, including pCAGGS-GP2–GP5, pCAGGS-M, pCAGGS-N, pCAGGS-nsp1α, pCAGGS-nsp1β, and pCAGGS-nsp2–nsp12. However, only plasmids encoding N, M, GP2–GP5, nsp1α, nsp1β, nsp2–nsp5, nsp7, and nsp9–nsp12 exhibited efficient expression. Subsequently, m^6^A dot blot assay was employed to detect the cellular m^6^A level in Marc-145 cells transfected with a plasmid carrying an nsp, N, M, or GP2–GP5 gene. The results showed that GP2–GP4, nsp3, nsp4, nsp9, nsp10, and nsp11 strongly increased the m^6^A level (Fig. 5*A*). Similar findings were also noted in RAW264.7 cells (Fig. 5*B*). Further investigation of the expression of m^6^A writers (METTL3 and METTL14) and erasers (AlkBH5 and FTO) by qRT-PCR and Western blot analysis revealed that the levels of METTL3 and METTL14 proteins were hardly influenced by PRRSV viral proteins in Marc-145 cells (Fig. 5*C*) and RAW264.7 cells (Fig. 5*D*). In contrast, the levels of AlkBH5 proteins were obviously impaired by GP2, GP4, GP5, nsp9, and nsp11, while the levels of FTO proteins were observably reduced by GP4, GP5, nsp9, and nsp11 (Fig. 5, *C* and *D*). Furthermore, GP2, nsp1α, and nsp1β increased METTL3 mRNA expression (Fig. 5, *E* and *F*); GP3 and nsp10 enhanced METTL14 mRNA expression (Fig. 5, *G* and *H*); GP5, nsp9, nsp11, and nsp12 decreased AlkBH5 mRNA expression (Fig. 5, *I* and *J*); and nsp7, nsp9, and nsp11 inhibited FTO mRNA expression (Fig. 5, *K* and *L*).

### PRRSV nsp9 upregulates IL-13 expression by accelerating degradation of m^6^A demethylase FTO mRNA

**Figure 6:**
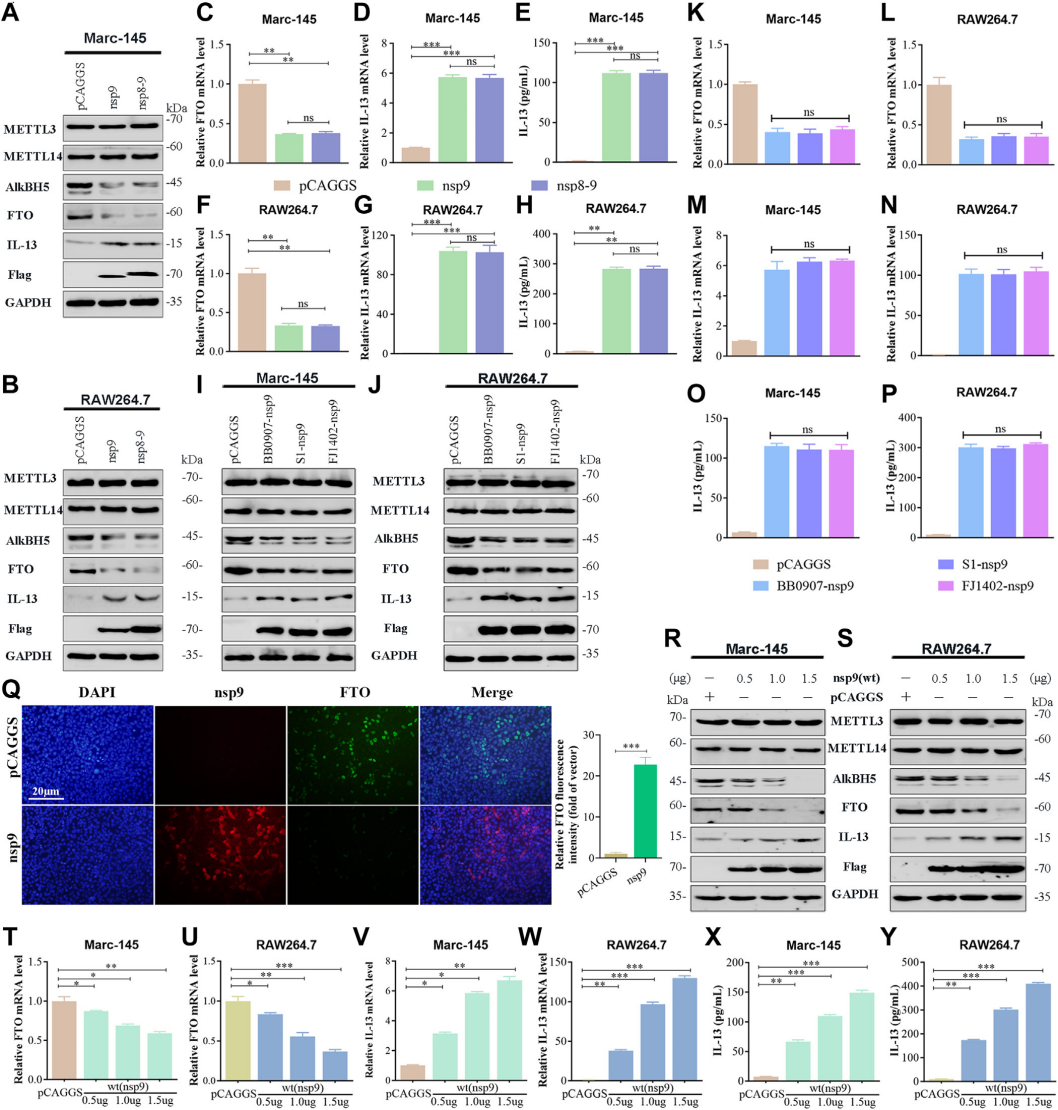
**PRRSV nsp9 upregulates IL-13 expression in a dose-dependent manner.***A*–*H*, transfection of Marc-145 and RAW264.7 cells with plasmids expressing nsp9, nsp8–9, or pCAGGS empty vector. *A* and *B*, Western blot analysis of the protein levels of METTL3, METTL14, AlkBH5, FTO, and IL-13. qRT-PCR analysis of the mRNA expression of (*C* and *F*) FTO and (*D* and *G*) IL-13. *E* and *H*, ELISA of IL-13 release. *I*–*P*, Marc-145 and RAW264.7 cells were transfected plasmids expression nsp9 gene from BB0907, S1, or FJ1402 strain for 48 h. *I* and *J*, Western blot analysis of the protein levels of METTL3, METTL14, AlkBH5, FTO, and IL-13. *K*–*N*, qRT-PCR analysis of the mRNA expression of (*K* and *L*) FTO and (*M* and *N*) IL-13. *O* and *P*, ELISA of IL-13 release. *Q*, IFA verification of the stable expression of FTO in RAW264.7 cells with or without transfection of nsp9. *R*–*Y*, Marc-145 and RAW264.7 cells transfected with PRRSV nsp9 at a dose of 0.5, 1.0, and 1.5 μg for 48 h. *R* and *S*, Western blot analysis of the protein levels of METTL3, METTL14, AlkBH5, FTO, IL-13, and PRRSV N protein. *T*–*W*, qRT-PCR analysis of the mRNA expression of (*T* and *U*) FTO and (*V* and *W*) IL-13. *X* and *Y*, ELISA of IL-13 release. Data represent mean ± SD of three independent experiments. ∗∗∗*p* < 0.001; ∗∗*p* < 0.01; ∗*p* < 0.05; ns, not significant. IL-13, Interleukin-13; METTL, methyltransferase-like; nsp9, nonstructural protein 9; PRRSV, porcine reproductive and respiratory syndrome virus.

As illustrated in Figure 5, nsp7, nsp9, and nsp11 inhibited FTO mRNA expression, while PRRSV nsp9 and nsp11 decreased FTO mRNA and protein expression. In particular, nsp9 caused maximum significant decrease in the FTO expression. However, as research on the functional mechanism of PRRSV nsp9 is limited, we first examined the effect of nsp9 on FTO-mediated IL-13 production. It has been reported that nsp8 serves as the N-terminal portion of nsp9. To analyze the relationship between nsp8 and nsp9 in the process of regulating FTO and IL-13 expression, we transiently expressed PRRSV nsp9 or nsp8–9 in RAW264.7 and Marc-145 cells and investigated the expression of FTO and IL-13 by qRT-PCR, Western blot analysis, and ELISA. The results obtained proved that both nsp9 and nsp8–9 similarly impaired the mRNA and protein levels of FTO in Marc-145 (Fig. 6, *A* and *C*) and RAW264.7 (Fig. 6, *B* and *F*) cells but enhanced IL-13 protein expression (Fig. 6, *A* and *B*), mRNA expression (Fig. 6, *D* and *G*), and secretion level (Fig. 6, *E* and *H*). Accordingly, we used the plasmid carrying only nsp9 gene in subsequent experiments. To examine the role of nsp9 from different PRRSV strains in stimulating IL-13 production, plasmids expressing the nsp9 gene from HP-PRRSV BB0907, Classical PRRSV S1, and NADC30-like strain FJ1402 were constructed. These plasmids were transfected in both Marc-145 cells and RAW264.7 cells. The results in Figure 6, *I–P* demonstrated that nsp9 from BB0907, S1, and FJ1402 strains equally stimulated the IL-13 production, as evidenced by the significant increase in IL-13 mRNA expression and secretion detected by qRT-PCR (Fig. 6, *M* and *N*) and ELISA (Fig. 6, *O* and *P*), respectively. While the FTO protein and mRNA levels were suppressed at the same level by nsp9 from all three strains of virus (Fig. 6, *I–L*). In addition, RAW264.7 cells were transfected with or without nsp9 plasmids. At 24 h after transfection, the cells were observed under confocal microscopy. Nsp9 inhibited FTO expression indicated by decreased intensity of the green fluorescence, while the expression of nsp9 shown with the red fluorescence (Fig. 6*Q*). The further experiments were performed in RAW264.7 and Marc-145 cells containing different concentrations of nsp9 construct, and the results revealed that nsp9 affects IL-13 production in a dose-dependent manner (Fig. 6, *R* and *S*, *V–Y*). Furthermore, the FTO protein and mRNA levels directly reduced with nsp9 concentrations (Fig. 6, *R*–*U*).

**Figure 7:**
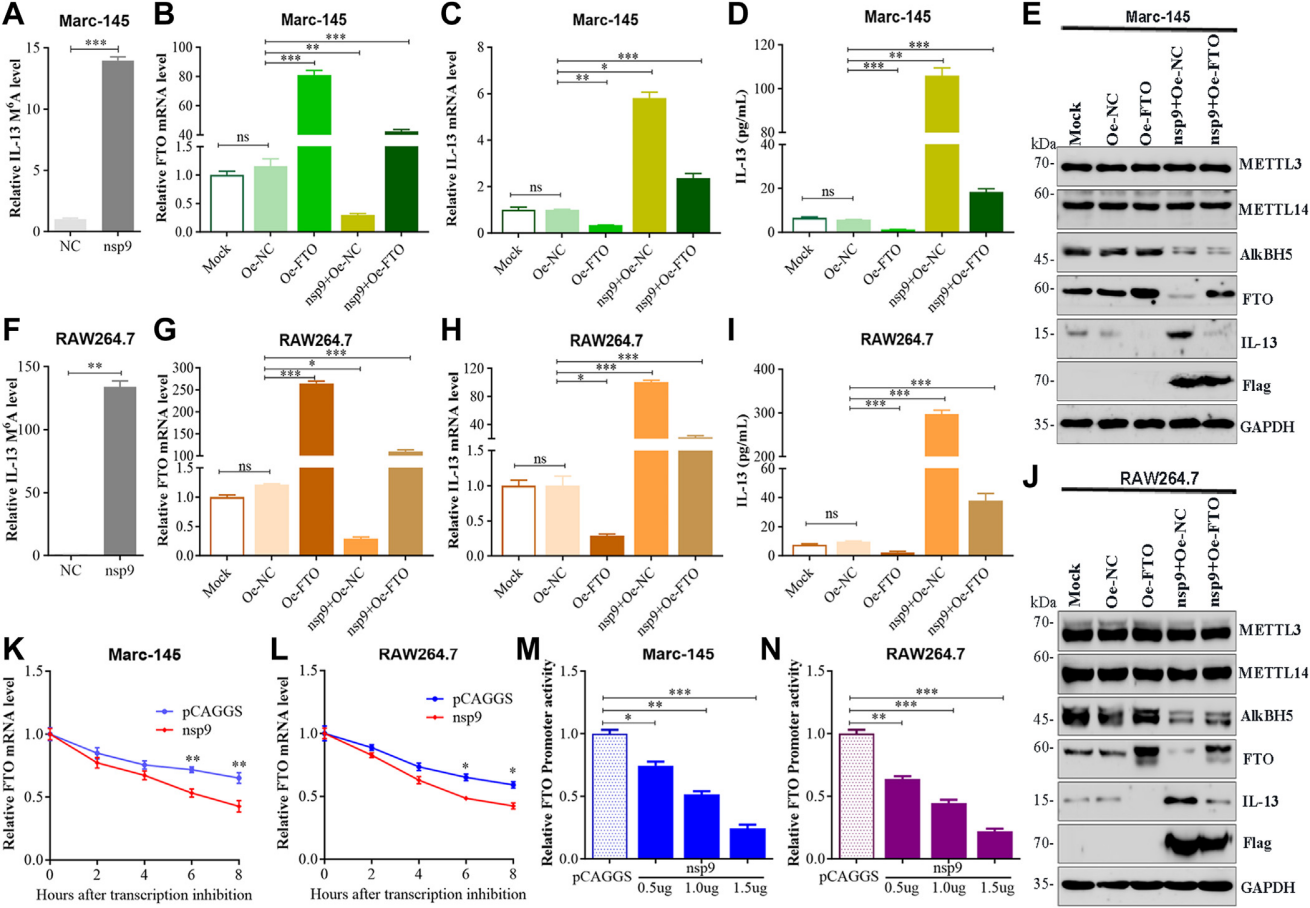
**PRRSV nsp9 upregulates IL-13 expression by modulating FTO mRNA stability and promoter.** Transfection of (*A*) Marc-145 and (*F*) RAW264.7 cells with plasmids expressing nsp9 or pCAGGS empty vector. IL-13 mRNA m^6^A level was determined by MeRIP-qPCR. *B*–*E* and *G*–*J*, Marc-145 and RAW264.7 cells transfected with Oe-NC or Oe-FTO with or without plasmid expressing nsp9. Untreated sample was used for calibration. qRT-PCR analysis of the mRNA expression of (*B* and *G*) FTO and (*C* and *H*) IL-13 in Marc-145 or RAW264.7 cells. ELISA of IL-13 release in (*D*) Marc-145 and (*I*) RAW264.7 cells. Western blot analysis of the lysates of transfected (*E*) Marc-145 and (*J*) RAW264.7 cells. *K* and *L*, lifetime of FTO mRNA in pCAGGS or nsp9 overexpression in Marc-145 or RAW264.7 cells. Relative mRNA levels were quantified by qPCR. *M* and *N*, the decrease in FTO promoter activity by nsp9 overexpression is dose-dependent in Marc-145 and RAW264.7 cells. Data represent mean ± SD of three independent experiments. ∗∗∗*p* < 0.001; ∗∗*p* < 0.01; ∗*p* < 0.05; ns, not significant. IL-13, Interleukin-13; m6A, N6-Methyladenosine; nsp9, nonstructural protein 9; PRRSV, porcine reproductive and respiratory syndrome virus.

We next explored if the modulation of IL-13 expression by nsp9 correlates with m^6^A levels. For this, total RNA from Marc-145 and RAW264.7 cells transfected with nsp9 for 48 h was isolated, and MeRIP-qPCR using IL-13 specific primers was performed. The results obtained proved that nsp9 was involved in m^6^A modulation of IL-13 expression (Fig. 7, *A* and *F*). To further confirm that FTO mediated the effect of nsp9-induced IL-13 expression, we conducted a functional rescue experiment by cotransfecting cells with nsp9 alongside Oe-NC or Oe-FTO. Compared to Mock and NC groups, the nsp9 group showed a substantial elevation in IL-13 expression and secretion (Fig. 7, *C*–*E* and *H–J*), with a concurrent significant decrease in FTO mRNA and protein levels (Fig. 7, *B*, *E*, *G* and *J*). FTO overexpression mitigated the nsp9-induced IL-13 upregulation in the nsp9+Oe-FTO group (Fig. 7, *C*–*E* and *H–J*), indicating that nsp9 enhances IL-13 production by downregulating FTO at both mRNA and protein levels. To decipher nsp9's role in FTO mRNA decay, overexpression studies in Marc-145 and RAW264.7 cells were conducted. mRNA stability assays revealed that nsp9 overexpression reduced the half-life of FTO mRNA in these cells (Fig. 7, *K* and *L*), suggesting nsp9-mediated regulation of FTO *via* mRNA stability. Additionally, to verify nsp9's involvement in FTO promoter activation, Marc-145 and RAW264.7 cells were cotransfected with pFTO-luc, pRL-TK, and varying concentrations of the pCAGGS-nsp9 plasmid. This revealed a dose-dependent decrease in FTO promoter activity with nsp9 concentrations (Fig. 7, *M* and *N*), further establishing nsp9's regulatory role.

### Truncated mutations in nsp9 attenuate the increase in IL-13 production

**Figure 8:**
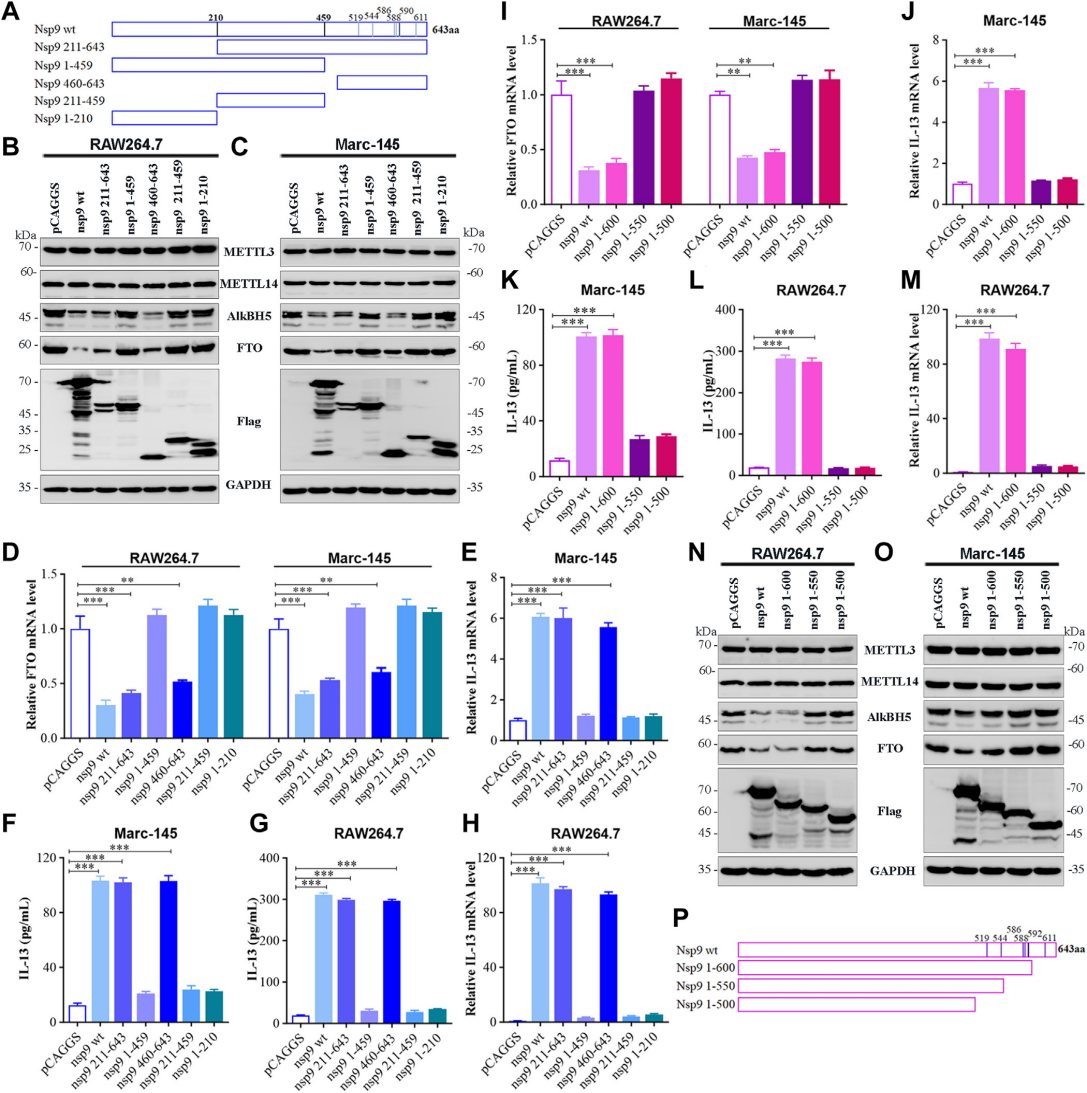
**Truncated mutations in nsp9 attenuate stimulation of IL-13 production.***A*, schematic representation of the nsp9(wt) and its truncated mutants. The mutants included those containing 1 to 643 aa, 1 to 459 aa, and 1 to 210 aa (encoding the N-terminus); 211 to 643 aa and 460 to 643 aa (encoding the C-terminus); and 211 to 459 aa. RAW264.7 and Marc-145 cells were transfected with nsp9(wt) or its truncated mutants. *B* and *C*, Western blot analysis of METTL3, METTL14, AlkBH5, FTO, and nsp9 (mutants) protein expression in (*B*) RAW264.7 and (*C*) Marc-145 cells. *D*, FTO mRNA level detected by qRT-PCR. *E* and *H*, IL-13 mRNA level determined by qRT-PCR and (*F* and *G*) IL-13 secretion ascertained by ELISA. RAW264.7 or Marc-145 cells were transfected with nsp9(wt) or a new truncated construct. qRT-PCR analysis of the mRNA level of (*I*) FTO and (*J* and *M*) IL-13. *K* and *L*, ELISA of IL-13 secretion. *N* and *O*, Western blot analysis of METTL3, METTL14, AlkBH5, FTO, and nsp9(mutants) protein expression in (*N*) RAW264.7 and (*O*) Marc-145 cells. *P*, schematic representation of the nsp9(wt) and its optimized truncated mutants. The mutants included 1 to 600, 1 to 550, and 1 to 500 aa (encoding the N-terminus). All data are representative of one of the three independent experiments. ∗∗∗*p* < 0.001; ∗∗*p* < 0.01; ∗*p* < 0.05. IL-13, Interleukin-13; METTL, methyltransferase-like; nsp9, nonstructural protein 9.

Truncated nsp9 mutants containing nucleotides 1 to 1929 (1–643 aa), 1 to 1377 (1–459 aa), and 1 to 630 (1–210 aa) (encoding the N-terminus); nucleotides 631 to 1929 (211–643 aa) and 1378 to 1929 (460–643 aa) (encoding the C-terminus); and nucleotides 631 to 1377 (211–459 aa) were derived from the intact nsp9 gene using PCR (Fig. 8*A*). The amplified products were inserted into a vector that could generate a chimeric polypeptide with a Flag tag at the C terminus. Subsequently, RAW264.7 or Marc-145 cells were transfected with pCAGGS-nsp9 or a truncated construct. The results of Western blot analysis showed no difference in the induction of FTO expression between the WT nsp9 (nsp9(wt)) and several mutants, including constructs containing 211 to 643 aa and 460 to 643 aa (Fig. 8, *B*–*D*). In contrast, the FTO protein level was significantly higher in the nsp9 mutant containing 1 to 459 aa, 1 to 210 aa, and 211 to 459 aa (Fig. 8, *B* and *C*). Furthermore, the FTO mRNA expression was increased (Fig. 8, D), whereas the IL-13 mRNA expression (Fig. 8, *E* and *H*) and secretion (Fig. 8, *F* and *G*) were decreased in the nsp9 mutant containing 1 to 459 aa, 1 to 210 aa, and 211 to 459 aa.

Subsequently, we sequentially constructed truncated nsp9 mutants containing nucleotides 1 to 1800 (1–600 aa), 1 to 1650 (1–550 aa), and 1 to 1500 (1–500 aa) (encoding the N terminus) with Flag tag into an expression vector pCAGGS (Fig. 8*P*) and transfected RAW264.7 or Marc-145 cells with pCAGGS-nsp9 or a new truncated construct. When compared with nsp9(wt), the nsp9 mutant containing 1 to 500 aa and 1 to 550 aa exhibited increased FTO mRNA and protein levels (Fig. 8, *I*, *N* and *O*) but decreased IL-13 production (Fig. 8, *J–M*). These results suggested that 551 to 600 aa in nsp9 contains a domain that stimulates IL-13 expression.

### Mutations in nsp9 reduce its ability to induce IL-13 production

**Figure 9:**
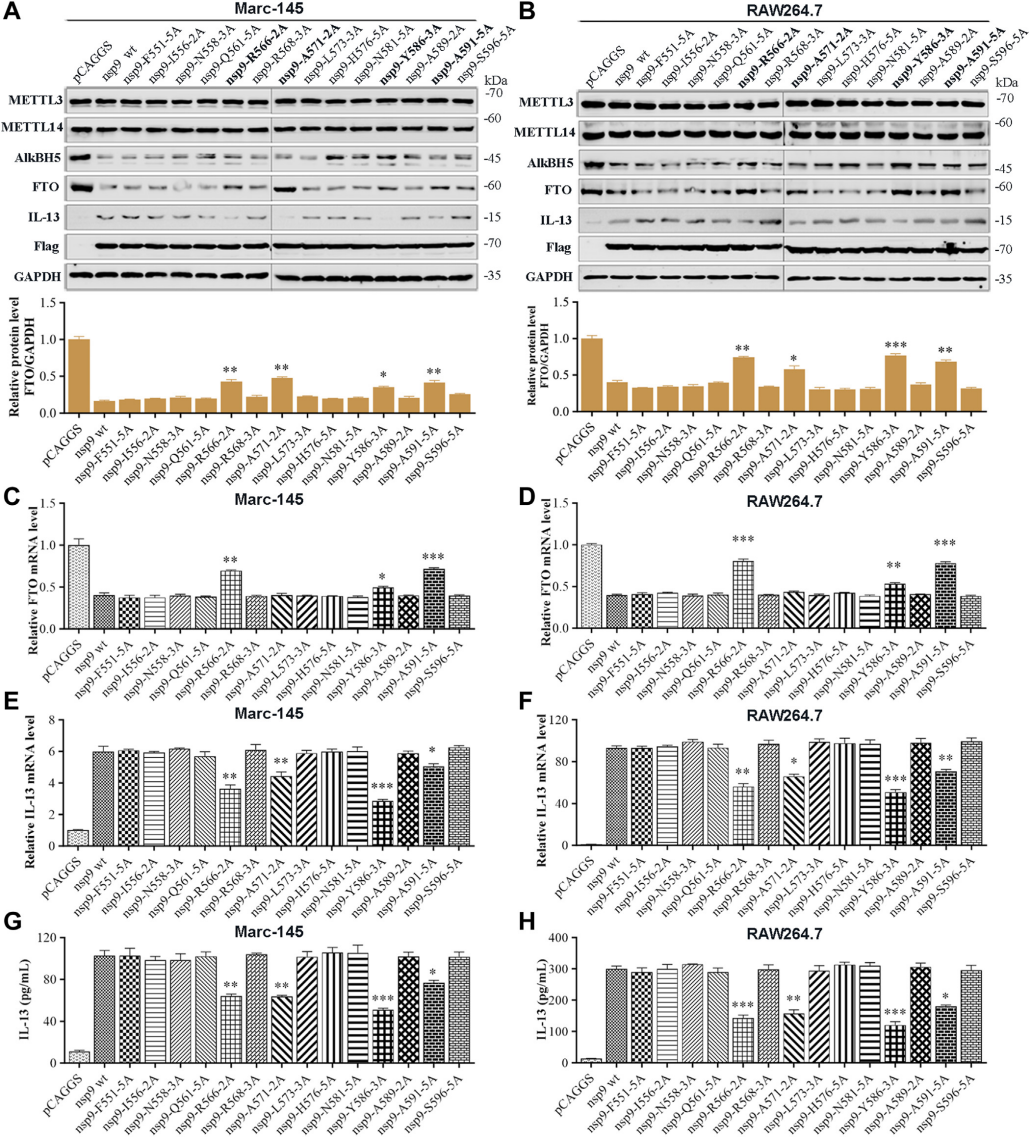
**Specific amino acid sites of nsp9 contribute to m**^**6**^**A demethylase FTO mediated IL-13 expression.***A*–*H*, transfection of nsp9(wt) or mutant expression plasmids into Marc-145 and RAW264.7 cells. Negative controls comprised cells transfected with pCAGGS. *A* and *B*, Western blot analysis of the protein level in cell lysates using relevant antibody. GAPDH was used as a loading control. *C*–*F*, qRT-PCR analysis of the mRNA expression of (*C* and *D*) FTO and (*E* and *F*) IL-13. *G* and *H*, ELISA of IL-13 secretion. All data were compared with the nsp9(wt) group. Data represent mean ± SD of three independent experiments. ∗∗∗*p* < 0.001; ∗∗*p* < 0.01; ∗*p* < 0.05. IL-13, Interleukin-13; m6A, N6-Methyladenosine; nsp9, nonstructural protein 9.

To refine the results of functional domain 551 to 600 aa in nsp9, we constructed 14 multipoint mutants with mutations in the putative functional region of nsp9 with the targeted amino acid residues replaced with alanine to obtain F551-5A, I556-2A, N558-3A, Q561-5A, R566-2A, R568-3A, A571-2A, L573-3A, H576-5A, N581-5A, Y586-3A, A589-2A, A591-5A, and S596-5A mutants (the nomenclature of the mutants indicates the location and number of alanine substitutions; *e.g.*, F551-5A indicates that five alanine substitutions were created beginning at position F551). As shown in Figure 9, *A*–*H*, the mutants R566-2A, A571-2A, Y586-3A, and A591-5A could restore inhibition of the FTO protein level in Marc-145 (Fig. 9*A*) and RAW264.7 (Fig. 9*B*) cells and lowered the increase in the levels of IL-13 protein (Fig. 9, *A* and *B*), mRNA (Fig. 9, *E* and *F*), and release (Fig. 9, *G* and *H*), when compared with the nsp9(wt) protein or any other multipoint mutants. Interestingly, when compared with nsp9(wt), only R566-2A, Y586-3A, and A591-5A mutants showed lower decrease in FTO mRNA level (Fig. 9, *C* and *D*), whereas A571-2A mutant did not show any difference.

**Figure 10:**
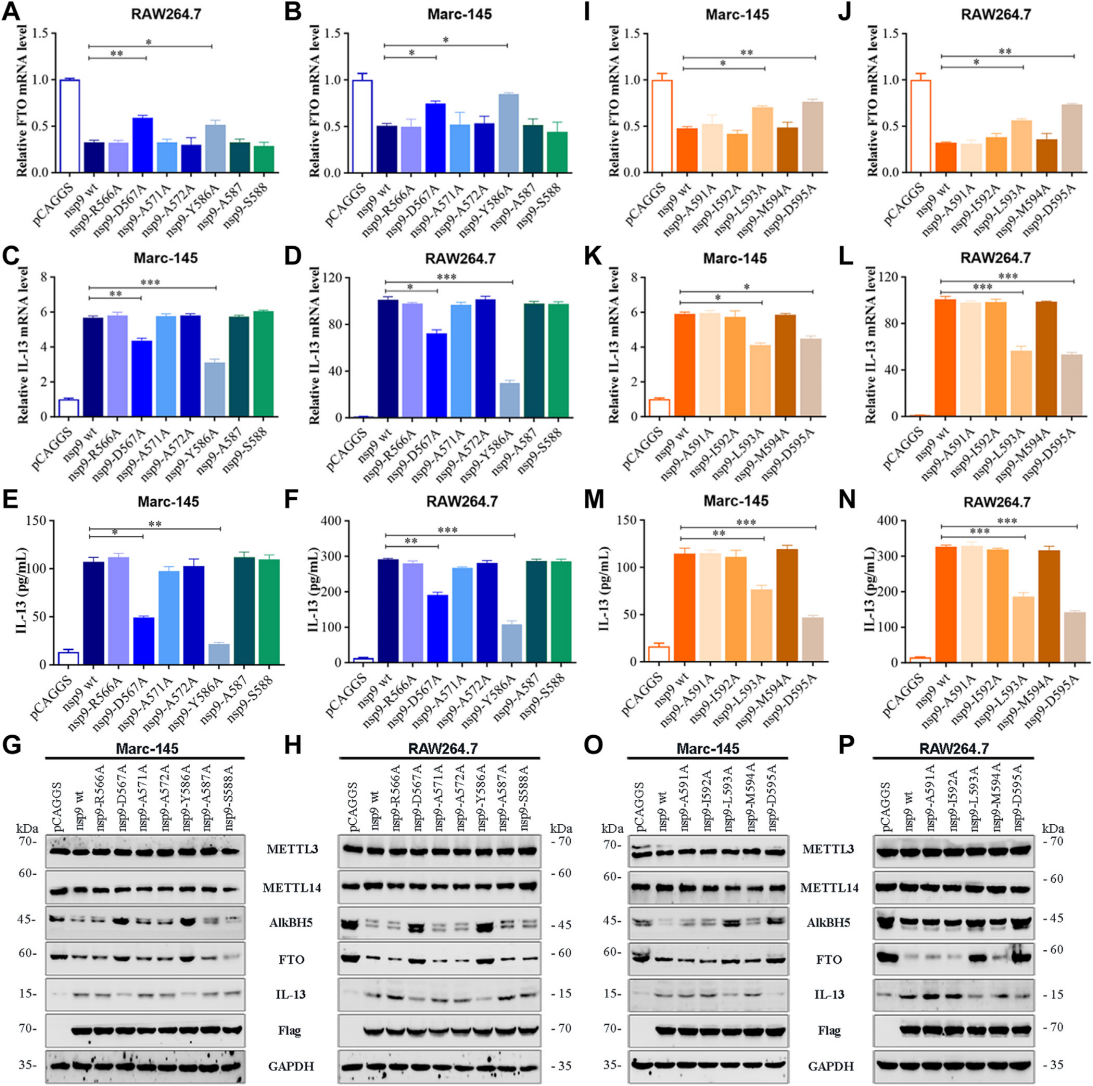
**Characterization of individual amino acid residues of nsp9 protein required for the induction of IL-13 expression *via* FTO degradation.** Marc-145 or RAW264.7 cells were transfected with plasmids expressing nsp9(wt) or a mutant protein (1 μg). Cells transfected with pCAGGS served as negative controls. *A*–*H*, individual alanine substitutions at positions 566, 567, 571, 572, and 586 to 588. qRT-PCR analysis of the mRNA expression of (*A* and *B*) FTO and (*C* and *D*) IL-13. *E* and *F*, ELISA of IL-13 secretion. *G* and *H*, Western blot analysis of protein level in cell lysates using relevant antibody. GAPDH was used as a loading control. *I*–*P*, individual alanine substitutions at positions 591 to 595. qRT-PCR analysis of the mRNA expression of (*I* and *J*) FTO and (*K* and *L*) IL-13. *M* and *N*, ELISA of IL-13 secretion. *O* and *P*, Western blot analysis of protein level in cell lysates using relevant antibody. GAPDH was used as a loading control. Data are expressed as mean ± SD, representative of three independent experiments. One-way ANOVA was used to determine statistical significance. ∗∗∗*p* < 0.001; ∗∗*p* < 0.01; ∗*p* < 0.05; ns, not significant. IL-13, Interleukin-13; nsp9, nonstructural protein 9.

To identify individual amino acid residues within the functional domains that are necessary for inducing IL-13 production, point mutations were constructed in the regions spanning 566 to 567 aa, 571 to 572 aa, 586 to 588 aa, and 591 to 595 aa. The mutations at Asp567, Tyr586, Leu593, and Asp595 significantly decreased IL-13 production (Fig. 10, *C–H* and *K–P*) and recovered FTO expression (Fig. 10, *A*, *B*, *I*, and *J*), when compared with the WT protein in Marc-145 and RAW264.7 cells. These findings indicated that Asp567, Tyr586, Leu593, and Asp595 are the functional domains necessary for the stimulation of IL-13 production. However, as each nsp9 mutant only comprised an amino acid mutation site, a single nsp9 mutation could not completely rescue change in FTO and IL-13 expression.

### The nsp9 mutant PRRSV plays a crucial role in m^6^A demethylase FTO-mediated IL-13 expression

**Figure 11:**
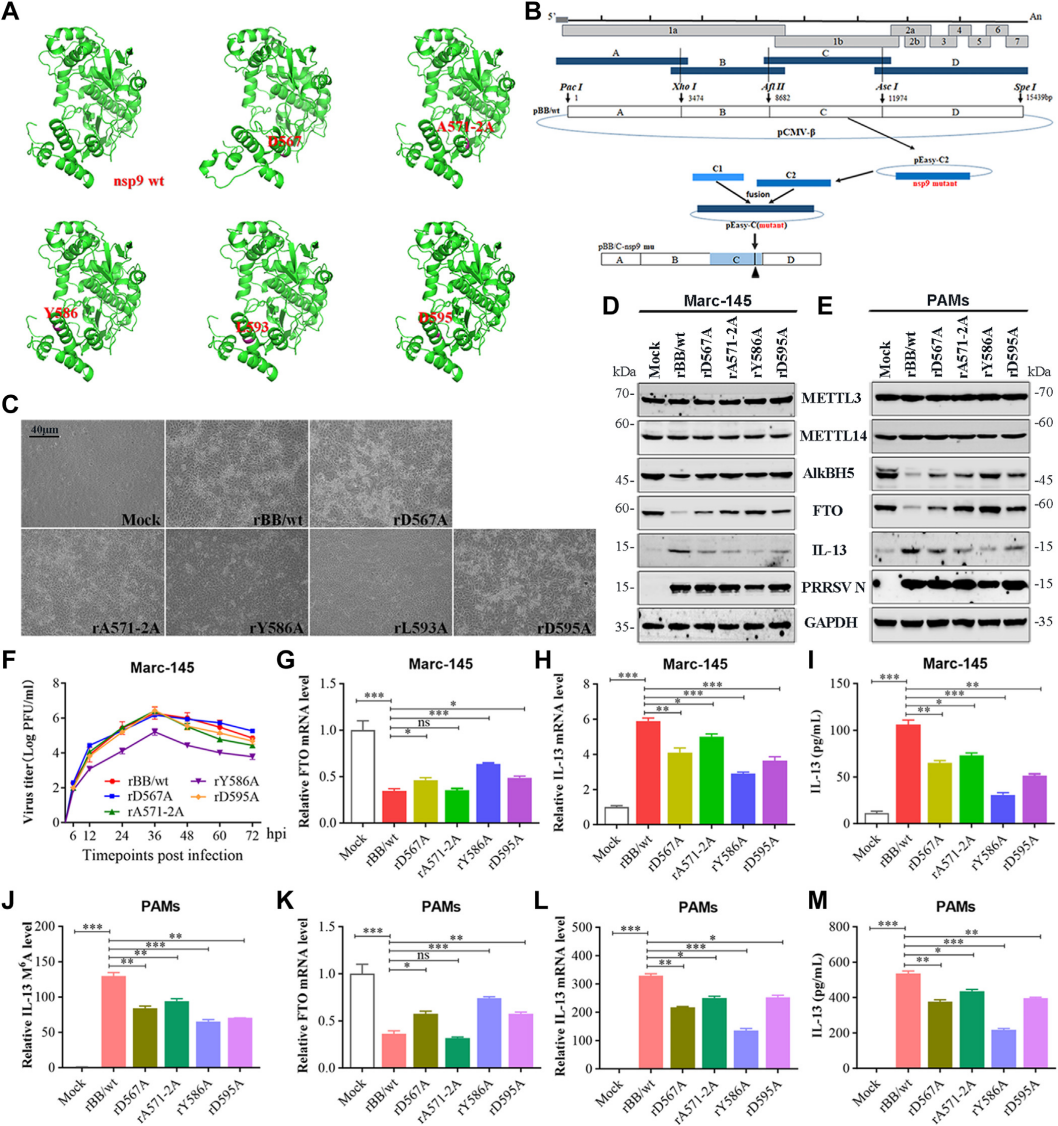
**Recombinant PRRSV strains containing nsp9 mutants increase FTO expression and decrease IL-13 expression.***A*, predicted three-dimensional structure of nsp9(wt) or nsp9 mutants of PRRSV strain BB0907, presented in cartoon formats. *B*, construction of full-length cDNA clones and the rescued PRRSV strains containing mutations in nsp9. *C*, Marc-145 cells were transfected with the full-length mutant cDNA clones pBB/D567A, pBB/YA571-2A, pBB/Y586A, pBB/L593A, and pBB/D595A. Light microscopy images of CPE were detected after 96 h. *D* and *E*, Western blot analysis of the protein expression of METTL3, METTL14, AlkBH5, FTO, N protein, and IL-13 in (*D*) Marc-145 or (*E*) PAMs. *F*, multistep growth kinetics of PRRSV in Marc-145 cells after infection with the indicated viral strain at an MOI of 0.1. Results are expressed as TCID_50_. *G*–*I*, Marc-145 cells inoculated with PRRSV rBB/wt, rD567A, rA571-2A, rY586A, or rD595A at an MOI of 1. qRT-PCR analysis of (*G*) FTO and (*H*) IL-13 mRNA expression. *I*, ELISA of IL-13 secretion. *J*–*L*, PAMs inoculated with PRRSV rBB/wt, rD567A, rA571-2A, rY586A, or rD595A at an MOI of 1. *J*, gene-specific m^6^A-RIP-qPCR analysis of the m^6^A levels in IL-13 mRNA transcript. *K* and *L*, qRT-PCR analysis of (*K*) FTO and (*L*) IL-13 mRNA expression. *M*, ELISA of IL-13 secretion. Data are mean ± SD of at least three biological replicates. ∗∗∗*p* < 0.001; ∗∗*p* < 0.01; ∗*p* < 0.05; ns, not significant. CPE, cytopathic effect; METTL, methyltransferase-like; MOI, multiplicity of infection; IL-13, Interleukin-13; m6A, N6-Methyladenosine; nsp9, nonstructural protein 9; PAM, porcine alveolar macrophage; PRRSV, porcine reproductive and respiratory syndrome virus; TCID_50_, 50% tissue culture infective dose; RIP, RNA immunoprecipitation.

The Asp567, Tyr586, Leu593, and Asp595 mutants exhibited impaired inhibition of FTO protein expression and lower IL-13 mRNA expression and secretion, when compared with nsp9(wt). Initially, we predicted the impact of mutations at Asp567, Tyr586, Leu593, and Asp595 on nsp9’s structure using SWISS-MODEL (https://swissmodel.expasy.org/). The predictions confirmed that these mutations did not alter the overall structural integrity (Fig. 11*A*). Then, to confirm the identities of the viral amino acids that play critical roles in the induction of IL-13 production, we attempted to obtain mutations affecting 567 aa, 571 to 572 aa, 586 aa, 593 aa, and 595 aa in nsp9 and constructed mutant viral strains containing mutations in nsp9 using PRRSV infectious cDNA clones (Fig. 11*B*). We successfully constructed full-length mutant cDNA clones pBB/D567A, pBB/YA571-2A, pBB/Y586A, pBB/L593A and pBB/D595A and transfected Marc-145 cells with each plasmid for 96 h to generate the mutant viruses rBB/wt, rD567A, rYA571-2A, rY586A, rL593A and rD595A. The mutant strains rD567A, rYA571-2A, rY586A, and rD595A were successfully rescued, which caused cytopathic effects in Marc-145 cells. Notably, the rY586A strain showed lower cytopathic effects than rBB/wt (Fig. 11*C*) and exhibited slower growth kinetics in early infection stages with about a 10-fold reduction in titers. The remaining mutants demonstrated growth kinetics similar to rBB/wt (Fig. 11*F*). Subsequently, Marc-145 cells infected with these mutants were analyzed *via* Western blot, ELISA, and qRT-PCR. The mutants D567A, A571-2A, Y586A, and D595A showed diminished FTO protein expression inhibition (Fig. 11*D*), reduced IL-13 protein level (Fig. 11*D*), mRNA expression (Fig. 11*H*), and secretion (Fig. 11*I*), whereas only rD567A, rY586A, and rD595A mutants showed FTO mRNA reduction (Fig. 11*G*), when compared with nsp9(wt). In addition, PAMs were also infected with these mutants at an MOI of 1 and examined by MeRIP-PCR. When compared with the rBB/wt-infected group, the IL-13 m^6^A level was remarkably decreased in the rD567A-, rA571-2A-, rY586A-, and rD595A-infected groups (Fig. 11*J*). qRT-PCR analysis showed that rD567A, rA571-2A, rY586A, and rD595A decreased the IL-13 mRNA level (Fig. 11*L*), whereas only rD567A, rY586A, and rD595A increased the FTO mRNA expression, when compared with rBB/wt (Fig. 11*K*). Moreover, the IL-13 secretion (Fig. 11*M*) and protein expression (Fig. 11*E*) in PAMs infected with rD567A, rA571-2A, rY586A, and rD595A were obviously lower than those in PAMs infected with the WT virus.

## Discussion

IL-13 is an important cytokine that plays crucial role in multiple immunoregulatory processes, contributing to cancer progression, tissue injury, respiratory disease, innate and adaptive antiviral immune response, and immunosuppression (23, 44, 45). It has been reported that IL-13 inhibited IFN-γ production, enhanced cytolytic potential, suppressed chemotactic migration of T lymphocytes, and stimulated IL-13 receptor a1 expression in T cells (46). During respiratory syncytial virus infection, elevated IL-13 induces and sustains long-term airway hyperreactivity and mucus production (47). Additionally, IL-13 levels are elevated in severe COVID-19 patients (48). IL-13–induced goblet cell metaplasia has been noted to contribute to airway remodeling and pathological mucus hypersecretion in asthma, and IL-13 has been found to promote susceptibility and abrogate acquired immunity to *Leishmania major* infection, possibly by acting as a Th2 cytokine and downregulating Th1 cytokines such as IFN-γ (30). Furthermore, IL-13 has been shown to significantly modulate respiratory syncytial virus infection, including decreasing the viral titers, inhibiting the lung IFN-γ production, and reducing weight loss, as well as affecting viral entry, replication, and cell-to-cell transmission (45). Besides, IL-13 has been observed to attenuate intense viral and cell shedding caused by SARS-CoV-2 infection by affecting viral entry, replication, and spread (44). In lung disease, high levels of IL-10 and IL-13 have been found to cause T- and B-cell–rich inflammatory response, subepithelial fibrosis, and mucus metaplasia with goblet cell hyperplasia and mucin hypersecretion, all of which may be the result of IL-10–induced IL-13 production (49). Many recent studies have indicated that various viral infections can induce IL-13 production and lead to pathological tissue damage through different mechanisms (24, 44, 46, 49, 50). We speculated that IL-13 might regulate inflammatory response and that chronic inflammation may cause lung damage and immune evasion during PRRSV infection. The results of the present study showed that PRRSV infection significantly increased IL-13 level in alveolar macrophages, suggesting that lung inflammation and injury were closely related to high level of IL-13 in PRRSV-infected pigs, considering the functions of IL-13 in inflammatory and immune responses.

Porcine reproductive and respiratory syndrome is characterized by disturbance of respiratory and genital systems in every growth stage of swine, causing significant loss to pig industry worldwide. PRRSV is a positive-strand RNA virus within the family Arteriviridae, and its genome comprises 15-kb nucleotides encoding eight structural proteins (GP2a, GP3, GP4, GP5, ORF5a, N, M, and E) and 16 nsp (nsp1α, nsp1β, nsp2, nsp2F/nsp2N, nsp3, nsp4, nsp5, nsp6, nsp7α, nsp7β, nsp8, nsp9, nsp10, nsp11, and nsp12) (51, 52, 53). Recent studies have revealed that PRRSV infection can modulate innate antiviral defense, cause immunosuppression, alleviate inflammatory response, and alter pathological reaction during lung damage. Besides, PRRSV infection can cause excessive secretion of proinflammatory cytokines and chemokines, including IL-1β, IL-4, IL-6, IL-8, IL-12, IL-17, and TNF-α (3, 12, 54, 55). The high level of IL-1β induced by PRRSV infection can significantly inhibit the replication and release of classical swine fever virus C strain proliferation. The induction of IL-1β by PRRSV is presumed to be mediated by TLR4–NF-κB–MAPK signaling pathways and NLRP3 inflammasome in host cells (3). PRRSV-2 has been noted to be involved in the regulation of lung injury by targeting STAT1 and TNF-α, which might be associated with mir-331-3p/mir-210 (6). Furthermore, HP-PRRSV infection has been observed to induce IL-6 expression, which could be associated with the activation of TAK-1–JNK–AP-1 and TAK-1–NF-κB signaling pathways (12).

PRRSV proteins play important roles in the expression of a variety of proinflammatory factors and are involved in the process of immune response, antiviral infection, immune evasion, and pathogenesis (11, 51, 52, 56, 57, 58). PRRSV infection can increase the HIF-1α levels, contributing to the development of inflammation and viral life cycle (11). One of the major properties of PRRSV nsp1β is the N-terminal nuclease activity and C-terminal deubiquitinating enzyme activity, while PRRSV nsp9 can downregulate PON1 to inhibit type-I IFN signaling pathway, eventually facilitating PRRSV replication (58). The Ser74 and Phe76 of PRRSV nsp11 can significantly induce IL-17 expression, which is also dependent on the activation of PI3K–p38MAPK–C/EBPβ/CREB pathways, thus causing alleviated lung inflammation and injury during PRRSV infection (55). Furthermore, through ubiquitination-proteasome pathway, PRRSV nsp6 can downregulate mucosa-associated lymphoid tissue lymphoma translocation protein 1 (MALT1), which plays essential roles in regulating immunity and inflammation, resulting in immune defense alleviation and virus survival (9). The further studies are necessary to determine the PRRSV proteins involved in IL-13 expression and the underlying mechanism.

In the present study, we focused on the function of m^6^A, which plays pivotal roles in the posttranscriptional regulation of gene expression and modulates multiple biological processes. Several studies have suggested that m^6^A is involved in virus–host interaction during viral infection (41, 43, 59, 60). HIV-1 has been noted to induce upregulation of m^6^A levels in cellular RNA *via* binding of HIV-1 gp120 to the CD4 receptor, which affects viral replication (61). Cellular m^6^A reader proteins (YTHDF2 and YTHDF3) machinery has been found to regulate the RIG-I signaling pathway activated by hepatitis C virus and HBV infection as a mechanism of immune evasion *via* m^6^A modification of viral RNA (62). Host cell m^6^A methyltransferase METTL3 has been reported to limit RIG-I binding to SARS-CoV-2 viral RNAs *via* additional m^6^A modifications and subsequent decrease in the activation of inflammation pathways (35). In the present study, we found that the expression of m^6^A demethylase FTO was decreased, while the level of m^6^A methylated RNA was elevated during PRRSV infection. Functional assays demonstrated that elevated m^6^A methylated RNA level and FTO suppression contributed to IL-13 secretion in Marc-145 cells and PAMs and that knockdown of FTO enhanced IL-13 gene expression, suggesting that higher cellular m^6^A levels may increase IL-13 secretion.

To identify viral proteins with the ability to stimulate IL-13 release, we constructed a series of recombinant plasmids by using an expression vector encoding GP2–GP5, N, M, and nsp proteins. Of these, only GP2–GP4, nsp3, nsp4, nsp9, and nsp11 significantly increased the m^6^A level. It must be noted that nsp9 contains an RNA-dependent RNA polymerase (RdRp) domain in the C terminus and a newly identified nidovirus RdRp-associated nucleotidyltransferase domain in the N terminus (63, 64). We focus on the functional aspects of the nsp9 C-terminal domain. The C-terminal RdRp domain is pivotal for viral RNA synthesis and replication efficacy. This domain’s interaction with the PRRSV N protein recruits cellular RNA helicases, facilitating the unwinding of the gRNA's double-stranded RNA structure, playing a significant role in viral RNA replication and transcription (65). Additionally, the leucine-rich repeat domain of NLRX1, which interacts with nsp9’s RdRp domain, is vital for its antiviral activity (66). The interaction between the DDX5 and nsp9 RdRp domains is also identified as a positive regulator of PRRSV replication (67). PRRSV nsp9 not only possesses polymerase activity but also interacts with other cellular proteins and PRRSV proteins, and investigation of the interaction of nsp9 with a zinc finger antiviral protein can provide insights into virus–host interaction (64). Recently study has revealed that genotype 2 PRRSV’s Nsp9 interacts with retinoblastoma protein, leading to retinoblastoma protein degradation *via* the ubiquitin-proteasome pathway in the cytoplasm (68). In addition, nsp9 can also interact with other proteins, including nucleotide-binding, oligomerization domain-like receptor (NLR) X1 annexin A2, retinoblastoma protein, and DEAD box RNA helicase 5 (63). Compelling evidences have demonstrated that m^6^A methylation plays critical roles in physiological processes, controlling RNA metabolism, stress response, and immune response *via* its “writer protein” (METTL3, METTL14) and “eraser protein” (FTO and AlkbH5) (43, 69, 70). FTO, as a demethylase protein of m^6^A RNA methylation, belongs to the ALKB family of Fe (II)/α-ketoglutarate-dependent dioxygenases, which may be regulated through multiple pathways and may participate in cell function (61). It has been reported that miR-149-3p repressed the expression of FTO gene through binding to the 3’-UTR of the FTO mRNA (69). During HIV infection, FTO has been noted to interact with cellular protein RIG-I, contributing to innate sensing of m^6^A-defective HIV-1 RNA in differentiated monocytic cells (43). Besides, the long noncoding RNA CASC15 has been observed to interact with FTO to regulate the progression of esophageal squamous cell carcinoma (70). To comprehensively understand the underlying mechanisms of these interactions, research on the interaction of nsp9 with FTO is necessary, which may be achieved using cellular proteins, microRNA, long non-coding RNA, etc. The present study revealed that nsp9 can significantly decrease the FTO mRNA and protein expression, largely owing to the nsp9-mediated mRNA decay. In addition, analysis of a sequence of recombinant plasmids expressing truncated nsp9 mutants revealed that 460 to 643 aa are important for the upregulation of m^6^A level and attenuation of FTO expression. Furthermore, 551 to 600 aa in nsp9 were found to play important roles in the stimulation of IL-13 production by reducing m^6^A demethylase FTO expression. Recent studies have shown that amino acids at positions 519, 544, 586, 588, 590, 592, 611, and 643 in nsp9 regulated the high virulence of PRRSV, interacted with other viral proteins, enhanced pathogenicity, and controlled cellular immune response (65, 71, 72, 73). The amino acid mutations at positions 519 and 544 of PRRSV nsp9 were found to be necessary for the rescue of PRRSV and critical to the replication efficiency of HP-PRRSV, contributing to enhanced pathogenicity (73). The amino acids at positions 586 and 592 in nsp9 have been reported to contribute to the replication efficiency of the Chinese HP-PRRSV in PAMs and act as critical sites determining the viral virulence in piglets (71). The amino acid residues at positions 588, 590, and 643 in nsp9 and at positions 62, 105, and 107 in nanobodies 6 (nb6) have been observed to be involved in nsp9–nb6 interaction, contributing to the inhibition of PRRSV replication (72). The E608 and E611 aa in nsp9 and Q85 aa in the N protein have been noted to be the pivotal residues involved in the N–nsp9 interaction, acting as anti-attenuation factors for the continuous elongation of nascent transcript during negative-strand RNA synthesis (65). In the present study, by using domain-specific mutations, we determined that the D567, Y586, L593, and D595 amino acid sites in nsp9 are important for the upregulation of IL-13 mRNA level and attenuation of FTO expression. Interestingly, when compared with nsp9(wt), 571 to 572 aa of nsp9 had no effects on FTO mRNA expression but restored suppression of FTO protein level. We presume that the modulatory effects of nsp9 on FTO mRNA or protein expression might be through different pathways, and the mechanism underlying PRRSV nsp9 interaction with FTO needs to be investigated in future studies.

Four mutant strains with mutations in nsp9 were rescued by using cDNA infectious clones, indicating that D567, A571-2A, and D595 are crucial for PRRSV-induced secretion of IL-13 through alteration of m^6^A demethylase FTO. PRRSV nsp9 has been reported to play a crucial role in viral replication, and the PRRSV nsp9 residues 586 and 592 are critical sites determining the viral replication efficiency and virulence (71). Accordingly, when compared with rBB/wt, rY586 prevented PRRSV replication and caused lower IL-13 secretion. As a Th2 family cytokine, IL-13 has been reported to play an important role in inflammation and lung tissue injury, with the ability to stimulate proliferation and survival of lymphocytes, achieve persistence and transformation of virus-infected T cells, and modulate inflammatory response microenvironment (18, 24, 74). Besides, IL-13 inhibition has been a known approach in immunotherapeutic treatments. Therefore, more effective vaccines against PRRSV could be developed in future by regulating the IL-13 level and immune response during PRRSV infection.

**Figure 12:**
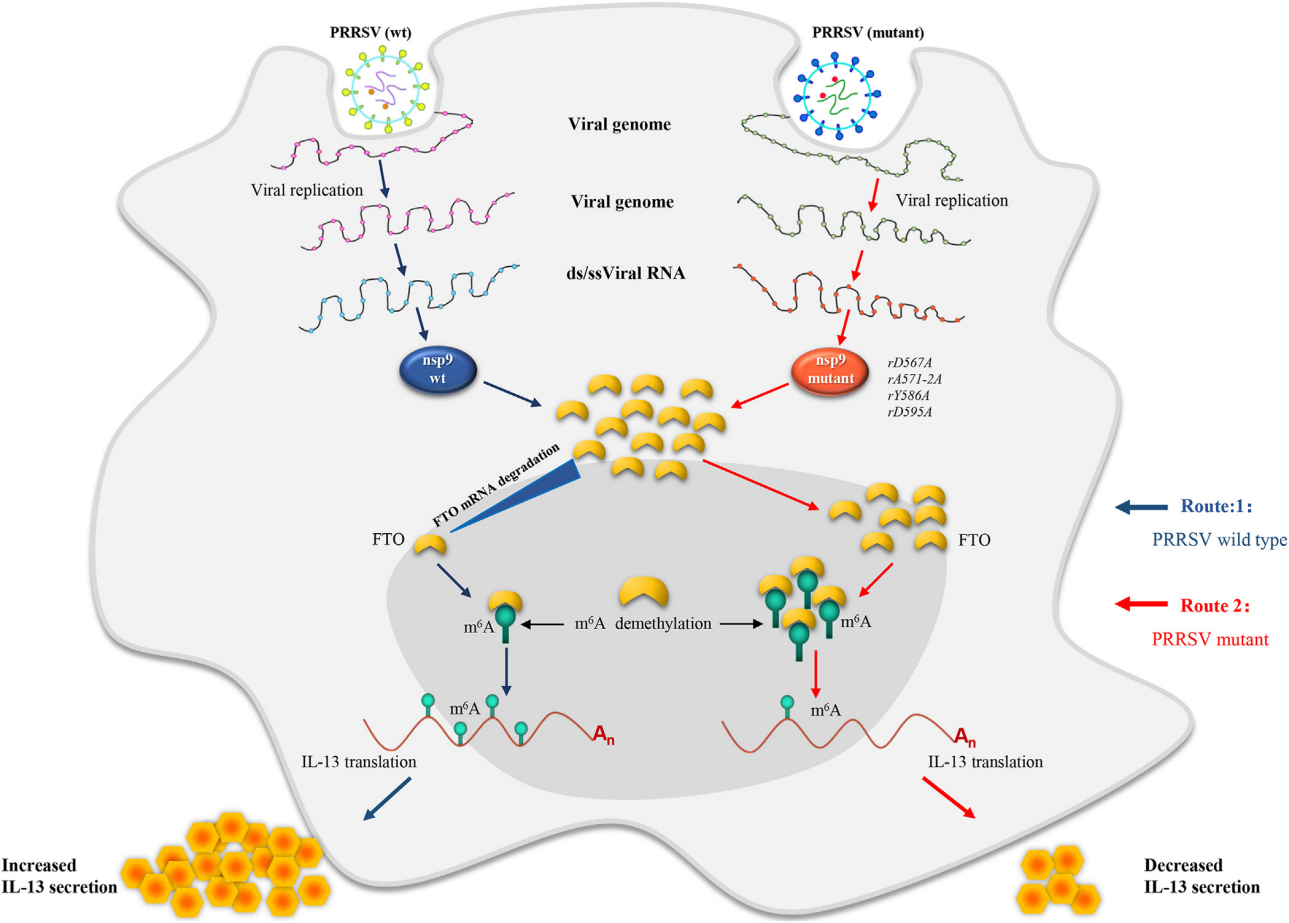
**Model demonstrating the ability of PRRSV nsp9 to induce IL-13 production mainly *via* FTO-bound m**^**6**^**A demethylation.** The model shows a possible mechanism by which PRRSV induces IL-13 production in PAMs. Following viral infection, intracellular accumulation of the viral protein, nsp9, inhibits FTO expression and then increases the m^6^A level, allowing it to bind to IL-13 m^6^A, thus increasing the release of IL-13. Mutations in nsp9 (rD567A, rA571-2A, rY586A, or rD595A) produce the opposite mechanism (*red arrow*). IL-13, Interleukin-13; m6A, N6-Methyladenosine; nsp9, nonstructural protein 9; PAM, porcine alveolar macrophage; PRRSV, porcine reproductive and respiratory syndrome virus.

In summary, the present study investigated the RNA modification profile of PRRSV, focusing on m^6^A modification and its functional relevance to the host cell immune response. The results revealed a new mechanism of PRRSV utilizing the host cellular m^6^A methyltransferase to modify its RNA and regulate the activation of downstream inflammatory genes. PRRSV infection was found to increase IL-13 expression in PAMs, which was predominantly owing to the increase in cellular m^6^A level with the attenuated expression of FTO. Besides, the m^6^A level was demonstrated to affect the IL-13 mRNA expression *via* FTO proteins. PRRSV nsp9 induced IL-13 secretion *via* acceleration of FTO mRNA degradation, and this function was specifically affected by D567, A572-2A, Y586, L593, and D595 amino acid sites of nsp9 (Fig. 12). These results highlighted the mechanism underlying the inflammatory and immune response regulated by cellular m^6^A machinery during viral infection.

## Experimental procedures

### Cells and virus

PRRSV has a highly restricted tropism for cells of the monocyte-macrophage lineage, especially PAMs, during acute infection of pigs. Currently, PRRSV can only be propagated *in vitro* in epithelial-derived Marc-145 cells, a subclone of the African green monkey kidney cell line. Primary PAMs and Marc-145 cells are generally used in virus infection experiments. The RAW264.7 cells belong to the mouse macrophage-like cell line, which have been widely used to evaluate macrophage-specific immune responses *in vitro*. Recent studies have constructed PRRSV-susceptible murine peritoneal macrophage-like RAW264.7 cell lines by achieving pCD163 cell surface expression in these cells (75), and the Viral titers in the RAW264.7^CD163^ cells were similar to those observed in primary PAMs. Moreover, PRRSV-induced cytokine expression patterns in RAW264.7^CD163^ cells closely mirrored the patterns observed in PAMs. Besides, many analyses on IL-13 have been frequently performed in RAW264.7 cells. Therefore, we used RAW264.7 cells in the present study (76, 77, 78).

PAMs were obtained from lung lavage of 8-week-old specific-pathogen-free pigs and cultured in RPMI 1640 medium (Gibco) containing 10% heat-inactivated fetal bovine serum (Gibco) and 1% penicillin-streptomycin (Gibco). Marc-145 cells (a monkey embryonic kidney epithelial cell line) and RAW264.7 cells (a mouse leukemia cells of monocyte macrophage) were obtained from the ATCC and grown in Dulbecco’s Modified Eagle’s Medium supplemented with 10% fetal bovine serum containing 1% penicillin-streptomycin in a 5% CO_2_ humidified atmosphere at 37 °C.

PRRSV strains used in this study comprised the HP-PRRSV strain BB0907 (GenBank Accession No. HQ315835) isolated in 2009 in Guangxi Province, China; classic PRRSV (C-PRRSV) strain S1 (GenBank Accession No. AF090173) isolated in 1997 from pigs with clinical signs of porcine reproductive and respiratory syndrome in Jiangsu Province, China; and low pathogenic PRRSV (LP-PRRSV) strain NT0801 (GenBank Accession No. HQ315836) isolated in 2008 in Jiangsu Province, China. Recombinant viruses (rBB/wt, rD567A, rA571-2A, rY586A, and rD595A) were rescued from infectious clone pCMV-BB0907 (the plasmid was kindly provided by Prof. Ping Jiang from the Institute of Nanjing Agricultural University, China). All the viruses were propagated in PAMs or Marc-145 cells, and the viral titers were determined using the Reed-Muench method and calculated as 50% tissue culture infective doses (TCID_50_).

### Animal experiment

Nine 4-week-old specific-pathogen-free piglets were randomly divided into three groups (three piglets per group). Three piglets were intranasally inoculated with 2 ml of PRRSV S1 or BB0907 strain(1 × 10^5^ TCID_50_ virus/ml). On day 10, we performed pathological dissection and collected all the lungs and PAMs of the piglets. The sacrificed pigs were removed, and the animal experiments were performed by random and blinded methods. This study was approved by the Jiangsu Normal University Animal Ethics Committee (protocol No. JSNUSK20221021-01).

### H&E stain

Lung tissue specimens from pigs were fixed in 4% paraformaldehyde solution for 24 h. Following fixation, the tissues underwent dehydration process and subsequently embedded in paraffin. Sections with a thickness of 4 μm were then prepared from these paraffin-embedded tissues. To assess lung pathology and inflammation, H&E staining was employed on these sections.

### Plasmid construction

The construction of Flag-tagged expression plasmids (pCAGGS-Flag) encoding PRRSV nsp1α, nsp1β, nsp2, nsp3, nsp4, nsp5, nsp7, nsp8-9, nsp9, nsp10, nsp11, nsp12, N, M, GP2, GP3, GP4, and GP5 was performed in our laboratory. The genes of nsp and structural proteins were amplified from HP-PRRSV strain BB0907 by RT-PCR using the primers listed in Table S1.

The truncated versions of nsp9 were subcloned from pCAGGS-nsp9 (including the Flag tag at the N terminus) and designated as pCAGGS-nsp9 (1–1377), pCAGGS-nsp9 (1–630), pCAGGS-nsp9 (631–1929), pCAGGS-nsp9 (1378–1929), pCAGGS-nsp9 (631–1377), pCAGGS-nsp9 (1–1800), pCAGGS-nsp9 (1–1650), and pCAGGS-nsp9 (1–1500). Alanine substitution mutations in the nsp9 genes were generated using PCR Phanta Super-Fidelity DNA Polymerase (Vazyme). All the constructs were confirmed by DNA sequencing, and the primers used for plasmid construction are presented in Table S2.

The whole PCR fragments were ligated into the pCAGGS vector (Invitrogen), generating an N-terminal Flag tag fusion plasmid. The protein expression of each constructed plasmid was detected by Western blot, analysis using anti-Flag antibody (Flag).

### siRNA transfection

The siRNA targeting ALKBH5 and FTO were utilized to silence AlkBH5 and FTO in cells, and a nontarget siRNA (si-NC)-loaded system was used as a control. The si-AlkBH5, si-FTO and si-NC were synthesized by BioSune (Shanghai). The primer sequences for si-AlkBH5, si-FTO, and si-NC are detailed in Table S3.

### Enzyme-linked immunosorbent assay

Supernatants from the cell cultures were harvested at designated times post infection. The IL-13 content in the supernatants was analyzed by using porcine IL-13 ELISA kit (RayBiotech) and human IL-13 ELISA kit (Abcam), following the manufacturers’ instructions.

### Western blot analysis

The levels of METTL3, METTL14, AlkBH5, FTO, IL-13, nsp9, N protein, and GAPDH were measured by Western blot analysis. In brief, the treated cells were collected and lysed on ice for 30 min in protein isolation buffer, subjected to SDS-PAGE, and transferred to nitrocellulose membrane. Then, the membrane was blocked with 10% low-fat milk at 37 °C for 4 h and probed with MAb anti-N (1:100; generously offered by Prof. Ping Jiang from the Institute of Nanjing Agricultural University), anti-FLAG (1:2000; Abmart), anti-METTL3 (1:1000; Proteintech), anti-METTL14 (1:1000; Proteintech), anti-AlkBH5 (1:1000; Proteintech), anti-FTO (1:1000; Proteintech), anti-IL-13 (1:1000; Proteintech), or anti-GAPDH (1:10,000; Abcam) at 37 °C for 1 h. After washing, the membrane was incubated with HRP-conjugated anti-mouse or anti-rabbit secondary antibody (1:1000; Abcam), and the bound proteins were visualized with a chemiluminescence (ECL; Biosharp) reagent.

### Real-time PCR

Quantitative real-time PCR was performed to measure the METTL3, METTL14, AlkBH5, FTO, N protein, IL-13, and GAPDH mRNA levels in PAMs, Marc-145, and RAW264.7 cells. The total RNA was extracted using Qiagen RNeasy kit (Qiagen) and cDNA was synthesized using an RT-PCR kit (TaKaRa), according to the manufacturers’ instructions. Quantitative real-time PCR was performed using SYBR Premix Ex Taq (TaKaRa), and the relative gene expression levels were determined using the 2^−ΔΔCt^ method and normalized to the housekeeping gene, GAPDH. The specific primer sequences used in this study are listed in Table S3.

### Construction of infectious cDNA clones of PRRSV

As shown in Figure 11*A*, a full-length cDNA clone (pCMV-BB) was obtained. The full-length PRRSV genome was amplified using the five primer pairs presented in Table S4 and employed as the backbone to construct nsp9 mutants in a PRRSV BB0907 background (51, 52). Mutagenesis of nsp9 was performed on the C fragment of pCMV-BB. Two plasmids containing the C fragment were constructed and labeled as pEASY-BB-C1 and pEASY-BB-C2, respectively. Site-directed mutations were accomplished using pEASY-BB-C2 as the template. Subsequently, splicing overlap extension PCR was performed using pEASY-BB-C1 and pEASY-BB-C2 (nsp9m) to construct the pEASY-BB-C (nsp9m) plasmid containing an nsp9 mutation. The AflII/AscI fragment of pCMV-BB was replaced with the corresponding region derived from the pEASY-BB-C (nsp9m) plasmid. The final full-length clone containing the mutation was labeled as pCMV-BB/nsp9m (Fig. 11*A*).

To rescue chimeric viruses, Marc-145 cells were transfected with pCMV-BB/nsp9m using Lipofectamine 3000 reagent according to the manufacturer’s protocol. The supernatants were harvested and serially passaged four times in Marc-145 cells, until about 80% of cells exhibited cytopathic effects. The P2–P5 virus stocks were prepared in the same manner. The rescued viruses were confirmed by whole-genome sequencing (data not shown) and labeled as rBB/wt, rD567A, rA571-2A, rY586A and rD595A.

### One-step viral growth curves

Marc-145 cells, seeded in 24-well plates, were inoculated with 10^6^ TCID^50^ of PRRSV. At 6, 12, 24, 36, 48, and 72 h post-infection (hpi), 100 μl of the infected cell supernatant were removed from each well and replaced with the same volume of fresh medium. All the samples were stored at −80 °C until virus titration, and the viral titers were determined as TCID_50_.

### Methylated RNA immunoprecipitation assay

Methylated RNA immunoprecipitation (MeRIP) assay was performed to examine m^6^A-modified IL-13. The total RNA from PAMs or Marc-145 cells was isolated using TRIzol reagent (Invitrogen), and 5 μg of anti-m^6^A antibody (Proteintech) or anti-rabbit IgG (Abcam) were conjugated to protein A/G magnetic beads. The eluted RNA was allowed to react with 200 μl of 0.5 mg/ml N6-methyladenosine 5-monophosphate sodium salt (Sigma-Aldrich) for 1 h at 4 °C. The immunoprecipitated RNA was reverse-transcribed to cDNA, and real-time PCR was subsequently performed with specific primers of IL-13.

### RNA immunoprecipitation assay

Enrichment of AlkBH5 or FTO in the IL-13 m^6^A region was further assessed using PureBindingRNA immunoprecipitation kit (Geneseed) according to the manufacturer’s instructions. In brief, the cells were lysed in RIP lysis buffer at 4 °C for 10 min, and the sample was divided into three parts (100 μl each), with two parts subjected to RIP with protein A/G sepharose beads conjugated with anti-AlkBH5 (5 μg, Proteintech) or anti-FTO (5 μg, Proteintech) antibody and normal rabbit IgG (Abcam) at 4 °C for 6 h. The RNA bound to the antibodies was purified and dissolved in RNASE-free water. RT-qPCR was employed to analyze the number of IL-13 transcripts in the immunoprecipitated RNA and total RNA from the whole cell lysates.

### The m^6^A dot blot assay

For the m^6^A dot blot assay, total RNA was extracted from cell aliquots using TRIzol, and the RNA samples were quantified by Nanodrop and UV crosslinked to nylon membranes. The membranes were blocked with 5% nonfat dry milk (in 1 × TBS) for 1 to 2 h and incubated with a specific anti-m^6^A antibody (1:2000, Proteintech) and mouse-HRP secondary antibodies. Then, the membranes were developed with ECL Western Blotting Substrate (Bio-Rad), visualized using Immobilon Western Chemilum HRP Substrate (Merck Millipore), washed once with TBST buffer for 5 min, and stained with MB for 30 min, followed by two or three washes with water. Subsequently, the relative m^6^A levels were determined by densitometry quantification and normalized to MB staining.

### mRNA stability analysis

Marc-145 and RAW264.7 cells were treated with 5 μg/ml actinomycin D (Sigma-Aldrich, A9415) to inhibit global mRNA transcription. After incubation for 0, 2, 4, and 8 h, mRNA was extracted from the cells and cDNA was synthesized using an RT-PCR kit (TaKaRa). The mRNA transcript levels of interest were detected by qPCR using primers specific to FTO.

### Statistical analysis

The data were obtained from at least three independent experiments for the quantitative analysis and are expressed as mean ± SD. GraphPad Prism (version 5.0; GraphPad Software) was used for data analysis, One-way ANOVA was utilized for comparison between multiple groups, following Tukey’s multiple comparison tests. The *t* test was employed to compare the average between the two groups.

## Data availability

The datasets used and/or analyzed during the current study are available from the corresponding author on reasonable request.

## Ethics statement

This study was approved by the Jiangsu Normal University Animal Ethics Committee (protocol No. JSNUSK20221021-01).

## Supporting information

This article contains supporting information (51, 52).

## Conflict of interest

The authors declare that they have no conflicts of interest with the contents of this article.
