# Supplementary material for: Highly pathogenic PRRSV upregulates IL-13 production through nonstructural protein 9–mediated inhibition of N6-methyladenosine demethylase FTO
Source: J Biol Chem. 2024 Mar 18;300(4):107199. doi: 10.1016/j.jbc.2024.107199 (PMC11017062; doi:10.1016/j.jbc.2024.107199)
Supplement: Supplemental Tables S1–S4 [file mmc1.docx]

**Table S1. Primers used to generate individual viral protein-expressing plasmids**

| **Primer** | **Sequence (5’-3’) ^a, b^** |
| --- | --- |
| Nsp1αF | ACTGAATTCATGTCTGGGATACTTGATCGGTGCA |
| Nsp1αR | TATCTCGAG**CTTATCGTCGTCATCCTTGTAATC**CTGCGGGAGCGGCAGGTTGGTCAAC |
| Nsp1βF | ACTGAATTCATGAGGCCCAAACCTGAGGACTTTTGCC |
| Nsp1βR | TATCTCGAG**CTTATCGTCGTCATCCTTGTAATC**GTACCACTTATGACTGCCAAACCGG |
| Nsp2F | TAAGGTACCATGGGTGCTGGAAAGAGAGCAAGAAGAA |
| Nsp2R | CACGCTAGC**CTTATCGTCGTCATCCTTGTAATC**GCCCAGTAACCTGCCAAGAATGGCA |
| Nsp3F | ACTGAATTCATGGGGGCACGCTACATCTGGCACTTTT |
| Nsp3R | TATCTCGAG**CTTATCGTCGTCATCCTTGTAATC**CTCAAGGAGGGACCCGAGCT |
| Nsp4F | ACTAAGCTTATG**G**GGCGCTTTCAGAACTCAAAAGCCCT |
| Nsp4R | TATCTCGAG**CTTATCGTCGTCATCCTTGTAATC**TTCCAGTTCGGGTTTGGCAGCAAGC |
| Nsp5F | ACTGAATTCGGAGGCCTTTCCACAGTTCAACTTC |
| Nsp5R | TATCTCGAG**CTTATCGTCGTCATCCTTGTAATCCTCGGCAAAGTATCGCAAGA** |
| Nsp7F | ACTATCGATATGTCGCTGACTGGTGCCCTCGCCATGA |
| Nsp7R | CACGCTAGC**CTTATCGTCGTCATCCTTGTAATC**TTCCCACTGAGCTCTTCTATTCTCG |
| Nsp9F | CGGGGTACCATGGTTTAAACTGCTAGCCGCCAGCGGC |
| Nsp9R | TATCTCGAG**CTTATCGTCGTCATCCTTGTAATC**CTCATGATTGGACCTGAGTTTTTCC |
| Nsp10F | CGGGGTACCATGGGGAAGAAGTCCAGAATGTGCGGGT |
| Nsp10R | CCGCTCGAG**CTTATCGTCGTCATCCTTGTAATC**TCATTCCAGGTCTGCGCAAAT |
| Nsp11F | ACTGAATTCTGGGGTCGAGCTCCCCGCTCCCCAAGG |
| Nsp11R | TATCTCGAG**CTTATCGTCGTCATCCTTGTAATC**TTTCAAGTTGAAAATAGGCCGTCTTG |
| Nsp12F | CGGGGTACCATGGGCCGCCATTTTACCTGGTATCAAC |
| Nsp12R | TATCTCGAG**CTTATCGTCGTCATCCTTGTAATC**ATTCAGGCCTAAAGTTGGTTCAATG |
| NF | CGGGGTACCCCAAATAATAACGGCAAGCAGCAAA |
| NR | TATCTCGAG**CTTATCGTCGTCATCCTTGTAATC**TCATGCTGAGGGTGATGCTG |
| MF | CGGGGTACCGGGTCGTCTCTAGACGACTTCTGCA |
| MR | TATCTCGAG**CTTATCGTCGTCATCCTTGTAATC**TTATTTGGCATATTTAACAA |
| Gp2F | CGGGGTACCAAATGGGGTCTATGCAAAGCCTCTT |
| Gp2R | TATCTCGAG**CTTATCGTCGTCATCCTTGTAATC**CCATGAGTTCAAAAGAAAAG |
| Gp3F | CGGGGTACCGCTAATAGCTGTACATTCCTCCATA |
| Gp3R | TATCTCGAG**CTTATCGTCGTCATCCTTGTAATC**TCGCCGTGCGGCACTGAGAA |
| Gp4F | CGGGGTACCGCTGCGCCCTTTCTTTTCCTCTTGG |
| Gp4R | TATCTCGAG**CTTATCGTCGTCATCCTTGTAATC**AATTGCCAGTAGGATGGCAA |
| Gp5F | CGGGGTACCTTGGGGAAGTGCTTGACCGCGTGCT |
| Gp5R | TATCTCGAG**CTTATCGTCGTCATCCTTGTAATC**GAGACGACCCCATTGTTCCG |

*a* The genomic positions for the primers are based on GenBank accession number HQ315835.

*b* The restriction enzyme sites used for cloning are underlined. The C-terminal FLAG tag in the R primer is indicated in boldface.

**Table S2. Primers used for construction of the nsp9 mutants**

| **Primer** | **Sequence (5’-3’)*^a^*** |
| --- | --- |
| nsp9F | CCCAAGCTTATGTTTAAACTGCTAGCCGCCAG |
| nsp9R | CCGCTCGAGTCACTTATCGTCGTCATCCTTGTAATCCTCATGATTGGACCTGAGTT |
| nsp9-631F | CCCAAGCTTATGGTGCCGACCATTCCAGCGTCCGTCC |
| nsp9-1378F | CCCAAGCTTATGTCAAACACCATTTACAGCTTAGTGA |
| nsp9-630R | CCGCTCGAGTCACTTATCGTCGTCATCCTTGTAATCATACAACTCAAAGCCAGAGG |
| nsp9-1377R | CCGCTCGAGTCACTTATCGTCGTCATCCTTGTAATCCACAGAGGTAATCGGGTCGC |
| nsp9-1500R | CCGCTCGAGTCACTTATCGTCGTCATCCTTGTAATCCTTGAGCATGTCCTCAAACT |
| nsp9-1650R | CCGCTCGAGTCACTTATCGTCGTCATCCTTGTAATCTGATGGTGAGTCTGTGATGG |
| nsp9-1800R | CCGCTCGAGTCACTTATCGTCGTCATCCTTGTAATCCACTGCGCTATCCCAACCAC |
| F551-5ASF | ACAGACTCACCATCA**TTCCTAGGTTGCAGG**ATAATAAATGGGCGC |
| F551-5ASR | GCGCCCATTTATTAT**CCTGCAACCTAGGAA**TGATGGTGAGTCTGT |
| I556-2ASF | TTCCTAGGTTGCAGG**ATAATA**AATGGGCGCCAGCTA |
| I556-2ASR | TAGCTGGCGCCCATT**TATTAT**CCTGCAACCTAGGAA |
| N558-3ASF | GGTTGCAGGATAATA**AATGGGCGC**CAGCTAGTCCCCAAC |
| N558-3ASR | GTTGGGGACTAGCTG**GCGCCCATT**TATTATCCTGCAACC |
| Q561-5ASF | ATAATAAATGGGCGC**CAGCTAGTCCCCAAC**CGTGACAGGATCCTC |
| Q561-5ASR | GAGGATCCTGTCACG**GTTGGGGACTAGCTG**GCGCCCATTTATTAT |
| R566-2ASF | CAGCTAGTCCCCAAC**CGTGAC**AGGATCCTCGCGGCC |
| R566-2ASR | GGCCGCGAGGATCCT**GTCACG**GTTGGGGACTAGCTG |
| R568-3ASF | CCCAACCGTGACAGG**AGGATCCTC**GCGGCCCTCGCCTAC |
| R568-3ASR | GTAGGCGAGGGCCGC**GAGGATCCT**CCTGTCACGGTTGGG |
| A571-2ASF | CGTGACAGGATCCTC**GCGGCC**CTCGCCTACCACATG |
| A571-2ASR | GTAGGCGAGGGCCGC**GAGGAT**CCTCCTGTCACGGTTGGG |
| L573-3ASF | AGGATCCTCGCGGCC**CTCGCCTAC**CACATGAAGGCAAGT |
| L573-3ASR | ACTTGCCTTCATGTG**GTAGGCGAG**GGCCGCGAGGATCCT |
| H576-5ASF | GCGGCCCTCGCCTAC**CACATGAAGGCAAGT**AATGTTTCTGAATAC |
| H576-5ASR | GTATTCAGAAACATT**ACTTGCCTTCATGTG**GTAGGCGAGGGCCGC |
| N581-5ASF | CACATGAAGGCAAGT**AATGTTTCTGAATAC**TACGCCTCGGCGGCT |
| N581-5ASR | AGCCGCCGAGGCGTA**GTATTCAGAAACATT**ACTTGCCTTCATGTG |
| Y586-3ASF | AATGTTTCTGAATAC**TACGCCTCG**GCGGCTGCAATACTC |
| Y586-3ASR | GAGTATTGCAGCCGC**CGAGGCGTA**GTATTCAGAAACATT |
| A589-2ASF | GAATACTACGCCTCG**GCGGCT**GCAATACTCATGGAC |
| A589-2ASR | GTCCATGAGTATTGC**AGCCGC**CGAGGCGTAGTATTC |
| A591-5ASF | TACGCCTCGGCGGCT**GCAATACTCATGGAC**AGCTGTGCTTGTTTA |
| A591-5ASR | TAAACAAGCACAGCT**GTCCATGAGTATTGC**AGCCGCCGAGGCGTA |
| S596-5ASF | GCAATACTCATGGAC**AGCTGTGCTTGTTTA**GAGTATGATCCTGAA |
| S596-5ASR | TTCAGGATCATACTC**TAAACAAGCACAGCT**GTCCATGAGTATTGC |

*a* The genomic positions of the primers were based on the sequence under GenBank accession number XM_001928655. The restriction enzyme sites used for cloning are underlined. The C- terminal Flag tag sequence in the “R” primers is indicated by double underlining. Locations of mutations are indicated in bold.

**Table S3. Sequence of primers for quantitative PCR analysis**

| **Name** | **Sequence (5’-3’)** |
| --- | --- |
| IL-13-(p) F | GGCAGTTTTCCTGCTTTCT |
| IL-13-(p) R | CAGTGGGGTCCACTCTCAAT |
| GAPDH-(p) F | CCTTCCGTGTCCCTACTGCCAAC |
| GAPDH-(p) R | GACGCCTGCTTCACCACCTTCT |
| IL-13-(h) F | CATACTCCAAACCTTTCCACCCC |
| IL-13-(h) R | TCAGCCCTCTTCAAAAACTTCTCCA |
| GAPDH-(h) F | AAGGCTGTGGGCAAGG |
| GAPDH-(h) R | TGGAGGAGTGGGTGTCG |
| IL-13-(m) F | CTGGCGGTGGCTCTCTTG |
| IL-13-(m) R | CCTTGGCAAAACTGCACCTT |
| GAPDH-(m) F | TCATGACCACAGTCCATGCC |
| GAPDH-(m) R | GGATGACCTTGCCCACAGCC |
| METTL3-(h) F | GAGATATGCTCTTAACCACCCG |
| METTL3-(h) R | GCTGCCCAATCCATCCAA |
| METTL14-(h) F | TATAAGGATGAACTAGAA |
| METTL14-(h) R | TGATGAGCTCCCTCAGTT |
| AlkBH5-(h) F | GCGCAGCGACTATGAGGA |
| AlkBH5-(h) R | GGCGGGTAGAGGCGCTCC |
| FTO-(h) F | GGTCGAGTTTGAGTGGCT |
| FTO-(h) R | AGGTAATGTTCGGGCAAT |
| METTL3-(p) F | CCATGATGGGTGCTGTGG |
| METTL3-(p) R | CTTCTTGCTCTGTTGTTC |
| METTL14-(p) F | ACAATCCTGGGAAGACTA |
| METTL14-(p) R | CCAGCCTGGTCGAATTGT |
| AlkBH5-(p) F | ACTCCGCGCTCTGCTTCG |
| AlkBH5-(p) R | GCTACCACGGAGCTGCTC |
| FTO-(p) F | AGATTGAGACCATCCAGG |
| FTO-(p) R | TTCCCCATGCCAAAGTAG |
| METTL3-(m) F | CCTACTCTTGTGACCTAT |
| METTL3-(m) R | GGCAGCATGTTTCCTTGA |
| METTL14-(m) F | TGGATGAAGGAGAGACAG |
| METTL14-(m) R | CTGTCAGCTAAACCTACA |
| AlkBH5-(m) F | TACACTTACGGCGCCCAG |
| AlkBH5-(m) R | GAAGCACAGCGCAGAGTC |
| FTO-(m) F | TAATGAGGTCGAGTTTGA |
| FTO-(m) R | TCATTCCTTTGTTCCACG |
| PRRSV N F | CCTCTAGCGACTGAAGATGACGTCAGGCATCACT |
| PRRSV N R | ACTCCACAGTGTAACTTATCCTCCCTGAATCT |

**Table S4. Primers used for construction of an infectious cDNA clone of strain BB0907**

| **Primer** | **Sequence (5’-3’) ^a, b^** |
| --- | --- |
| A-BB0907-Fwd | GCGTTAATTAAACCGTCATGACGTATAGGTGTTG |
| A-BB0907-Rev | TGTCTCGAGAATCATCTTTGGGAGAAACC |
| B-BB0907-Fwd | TTCTTAATTAAATGATTCTCGAGACACCGCC |
| B-BB0907-Rev | GTGCTTAAGTTCATTACCACCTGTAACGGAT |
| C1-BB0907-Fwd | GCGTTAATTAAAATGAACTTAAGCACCTATGCC |
| C1-BB0907-Rev | TTGACACAGAGGTAATCGGGTCGCCAGAC |
| C2-BB0907-Fwd | GTCTGGCGACCCGATTACCTCTGTGTCAA |
| C2-BB0907-Rev | CGGGGGAAAATGAAACCTCATGCTGGT |
| D1-BB0907-Fwd | TCGTTAATTAAGTTTCGGGCGCGCCAGAAAGGG |
| D-BB0907-Rev(SwaI) | TTCGGCTTGGGATTTAAATATGCATTTTTTTTTTTTTTTTTTTTT |
| D-BB0907-Rev(SpeI) | CTCACTAGTAACGGCCGCCAGTGTGCTGGAATTCGGCTTGGGATTT |
| XhoI-BB0907-Fwd | GGTACCATGGCCAAACTCGAGGCTTTTGCCGATACC |
| XhoI-BB0907-Rev | GGTATCGGCAAAAGCCTCGAGTTTGGCCATGGTACC |

*a* The genomic positions for the primers are based on GenBank accession number HQ315835.

*b* The restriction enzyme sites used for cloning are underlined.
